# EPHB6 augments both development and drug sensitivity of triple-negative breast cancer tumours

**DOI:** 10.1038/s41388-018-0228-x

**Published:** 2018-04-27

**Authors:** Behzad M. Toosi, Amr El Zawily, Luke Truitt, Matthew Shannon, Odette Allonby, Mohan Babu, John DeCoteau, Darrell Mousseau, Mohsin Ali, Tanya Freywald, Amanda Gall, Frederick S. Vizeacoumar, Morgan W. Kirzinger, C. Ronald Geyer, Deborah H. Anderson, TaeHyung Kim, Alana L. Welm, Peter Siegel, Franco J. Vizeacoumar, Anthony Kusalik, Andrew Freywald

**Affiliations:** 1 Department of Pathology and Laboratory Medicine, College of Medicine, University of Saskatchewan, Room 2841, Royal University Hospital, 103 Hospital Drive, Saskatoon, SK S7N 0W8 Canada; 2 Faculty of Science, Damanhour University, Damanhour, 22516 Egypt; 3 Department of Computer Science, University of Saskatchewan, 176 Thorvaldsen Bldg., 110 Science Place, Saskatoon, SK S7N 5C9 Canada; 4 Department of Chemistry and Biochemistry, Faculty of Science, University of Regina, Room 232, Research and Innovation Centre, Regina, SK S4S 0A2 Canada; 5 Cell Signaling Laboratory, Department of Psychiatry, College of Medicine, University of Saskatchewan, GB41 Health Sciences Building, 107 Wiggins Road, Saskatoon, SK S7N 5E5 Canada; 6 Department of Biochemistry, College of Medicine, University of Saskatchewan, Room 2D01 Health Science Building, 107 Wiggins Road, Saskatoon, SK S7N 5E5 Canada; 7 Saskatchewan Cancer Agency, University of Saskatchewan, 4D30.2 Health Sciences Building, 107 Wiggins Road, Saskatoon, SK S7N 5E5 Canada; 8 Donnelly Centre for Cellular and Biomolecular Research and Department of Computer Science, University of Toronto, Toronto, ON M5S 3E1 Canada; 9 Department of Oncological Sciences, Huntsman Cancer Institute, University of Utah, 2000 Circle of Hope, Salt Lake City, UT 84112 USA; 10 Goodman Cancer Research Centre, McGill University, 1160 Pine Avenue West, Montreal, QC H3A 1A3 Canada

## Abstract

Triple-negative breast cancer (TNBC) tumours that lack expression of oestrogen, and progesterone receptors, and do not overexpress the HER2 receptor represent the most aggressive breast cancer subtype, which is characterised by the resistance to therapy in frequently relapsing tumours and a high rate of patient mortality. This is likely due to the resistance of slowly proliferating tumour-initiating cells (TICs), and understanding molecular mechanisms that control TICs behaviour is crucial for the development of effective therapeutic approaches. Here, we present our novel findings, indicating that an intrinsically catalytically inactive member of the Eph group of receptor tyrosine kinases, EPHB6, partially suppresses the epithelial–mesenchymal transition in TNBC cells, while also promoting expansion of TICs. Our work reveals that EPHB6 interacts with the GRB2 adapter protein and that its effect on enhancing cell proliferation is mediated by the activation of the RAS-ERK pathway, which allows it to elevate the expression of the TIC-related transcription factor, OCT4. Consistent with this, suppression of either ERK or OCT4 activities blocks EPHB6-induced pro-proliferative responses. In line with its ability to trigger propagation of TICs, EPHB6 accelerates tumour growth, potentiates tumour initiation and increases TIC populations in xenograft models of TNBC. Remarkably, EPHB6 also suppresses tumour drug resistance to DNA-damaging therapy, probably by forcing TICs into a more proliferative, drug-sensitive state. In agreement, patients with higher EPHB6 expression in their tumours have a better chance for recurrence-free survival. These observations describe an entirely new mechanism that governs TNBC and suggest that it may be beneficial to enhance EPHB6 action concurrent with applying a conventional DNA-damaging treatment, as it would decrease drug resistance and improve tumour elimination.

## Introduction

EphA (EPHA1–EPHA8 and EPHA10) and EphB (EPHB1–EPHB4 and EPHB6) receptors comprise the largest group of receptor tyrosine kinases (RTKs) in human tissues. Their ligands, ephrins, are divided into A and B classes based on structural properties: ephrin-As (ephrin-A1–ephrin-A5) are GPI-anchored cell membrane proteins, and ephrin-Bs (ephrin-B1–ephrin-B3) display transmembrane and cytoplasmic domains. Ephrin binding induces tyrosine phosphorylation of Eph receptors, which enhances their catalytic activity and potentiates interactions with cytoplasmic partners, allowing for the control of a complex array of signalling pathways [1, 2]. Interestingly, both EphA and EphB groups possess kinase-deficient members, EPHA10 and EPHB6, suggesting that these molecules may have a crucial role in modulating biological outputs in the Eph receptor network [1]. Through their basal or ligand-induced signalling, kinase-active Eph receptors are frequently implicated in enhancing malignant behaviour of cancer cells [3] and in controlling tumour-initiating cells (TICs) [4]. In contrast, a strong negative correlation exists between the aggressiveness of solid tumours and kinase-dead EPHB6, with EPHB6 expression frequently reduced in aggressive malignancies, including invasive melanoma [5], metastatic lung and colorectal cancers [6], aggressive neuroblastoma [7, 8], prostate, gastric and ovarian tumours [9–11]. EPHB6 also suppresses metastasis in xenograft models of human lung cancer [12], melanoma [13] and colorectal cancer [14], while our previous work indicates that it undergoes tyrosine phosphorylation in breast cancer cells and inhibits breast cancer invasiveness [15]. Despite accumulating evidence, suggesting an important tumour-suppressing role for EPHB6, our understanding of its function in malignancy is far from complete. Here, we discuss our novel findings, describing a complex and intriguing action of EPHB6 in controlling the initiation, growth and drug resistance of triple-negative breast cancer (TNBC) tumours that lack the oestrogen receptor (ER), progesterone receptor (PR), do not overexpress the HER2 receptor, and represent the most aggressive breast cancer type [16].

## Results

### EPHB6 expression is reduced in breast cancer tumours, but is better preserved in TNBC

**Fig. 1:**
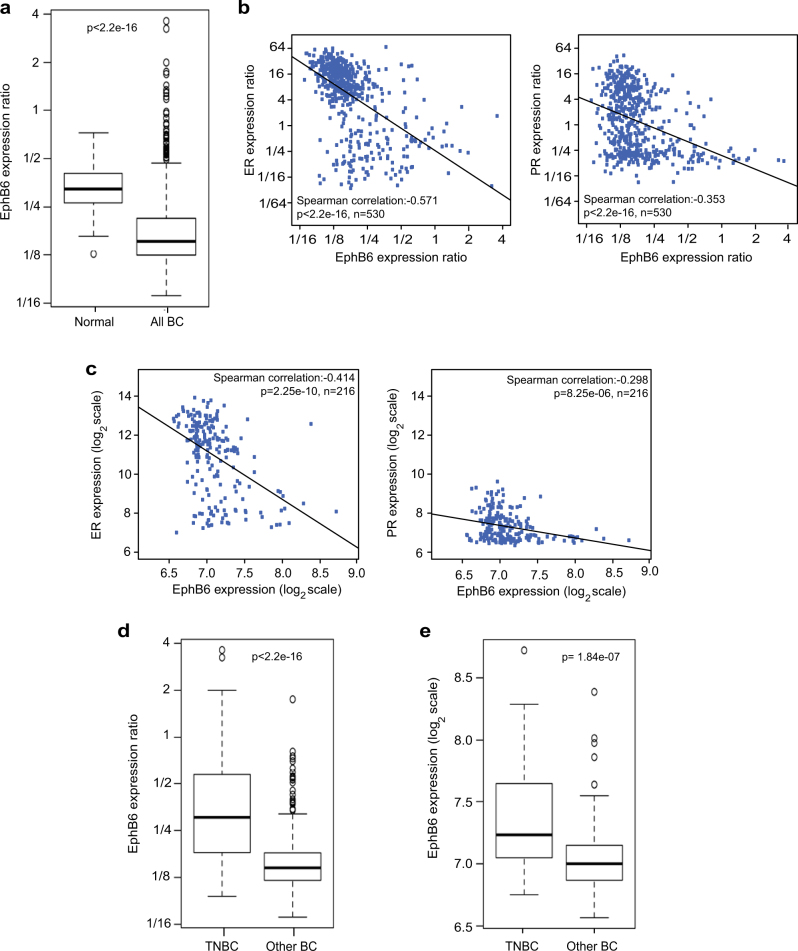
Expression of the EPHB6 receptor in breast cancer. **a** EPHB6 expression in 530 independent breast cancer samples (All BC) and 62 matching normal samples (Normal) was analysed using The Cancer Genome Atlas (TCGA) database. EPHB6 expression levels are provided as a log_2_ ratio to EPHB6 expression in Stratagene Universal Human Reference RNA. Statistical significance was determined by the Wilcoxon rank-sum test. Outliers consist of 47 samples, where 34 samples are of triple-negative origin and 13 represent other breast cancer subtypes. **b**, **c** Data from 530 breast cancer samples from TCGA and 216 samples from the European Institute of Bioinformatics (EBI) ArrayExpress database were analysed for EPHB6, oestrogen receptor (ER) and progesterone receptor (PR) expression. The scatterplots represent correspondence of EPHB6 expression with ER and PR in TCGA (**b**) and EBI (**c**). Expression values in **b** are in the same units as in **a**. Expression levels in **c** are log_2_ intensities. Included are Spearman correlations and lines showing the linear fit. **d** TCGA gene expression microarray data from 102 triple-negative tumours (TNBC) and 428 tumours of other breast cancer subtypes (Other BC) were analysed for EPHB6 expression. Expression values are presented as in **a**. *P*-value was computed by the Wilcoxon rank-sum test. **e** EBI gene expression microarray data from 40 triple-negative tumours and 176 tumours of other breast cancer subtypes were analysed for EPHB6 expression. Expression values are presented as in **c** and *P*-value calculated using the Wilcoxon rank-sum test

While EPHB6 expression is reduced in invasive breast cancer cell lines [17, 18], little is known about EPHB6 behaviour in breast cancer tumours. To fill this knowledge gap, we analysed the TCGA gene expression database, assessing EPHB6 status in 530 tumours and 62 normal samples. Our investigation revealed that EPHB6 abundance is significantly reduced in breast cancer (Fig. 1a), which expanded on previous observations that relied solely on breast cancer cell lines. Unexpectedly, our work with the TCGA and European Bioinformatics Institute (EBI) ArrayExpress datasets [19] showed that EPHB6 expression negatively correlates with the expression of ER and PR (Fig. 1b, c), suggesting that it might be better maintained in TNBC. Indeed, we found that EPHB6 expression was significantly better preserved in TNBC tumours (Fig. 1d, e) and a similar trend was also observed in breast cancer cell lines, although it did not achieve a statistical significance there, most probably because EPHB6 levels became more variable in the absence of the selective pressure of tumour microenvironment (Supplementary Figure S1A). Taken together, these data implied that EPHB6 may have a prominent role in the biology of TNBC.

### EPHB6 suppresses EMT in TNBC cells

**Fig. 2:**
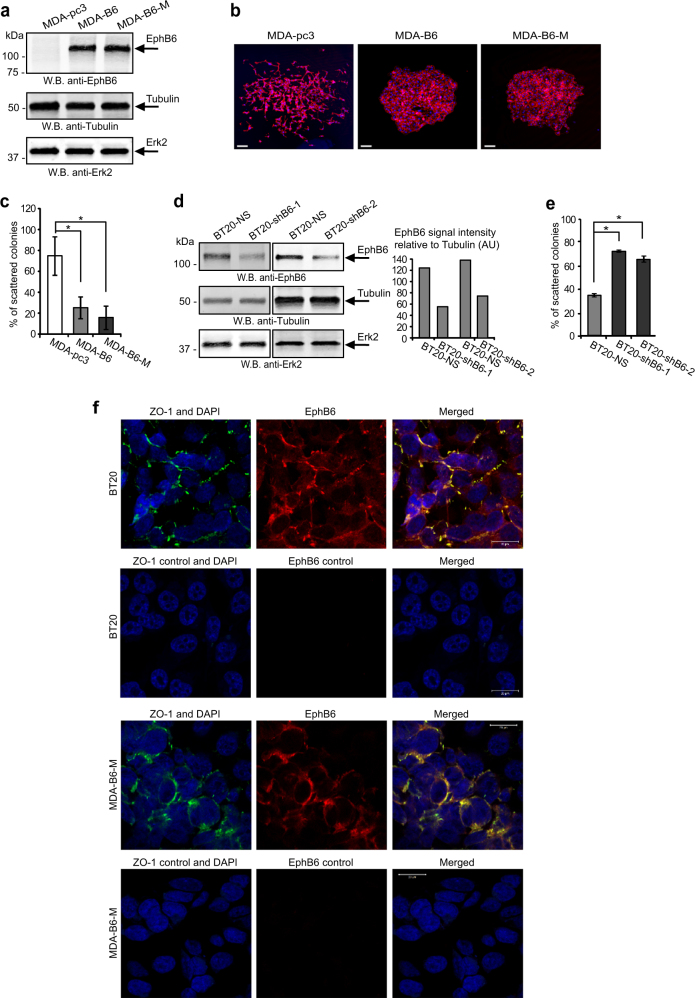
EPHB6 inhibits scattering of TNBC cells. **a** EPHB6-deficient TNBC cells, MDA-MB-231, were stably transfected with expression vectors encoding EPHB6 (MDA-B6), myc-tagged EPHB6 (MDA-B6-M), or mock-transfected with an empty vector (MDA-pc3). EPHB6 expression was analysed by Western blotting; tubulin and ERK2 represent loading controls. **b** MDA-pc3, MDA-B6 and MDA-B6-M cells were grown in individual colonies on glass coverslips in 24-well plates. Colonies were fixed and stained with phalloidin (red) and DAPI (blue). Representative colonies were imaged using an Olympus FV1000 confocal microscope with a 10× objective lens. Scale bar, 100 µm, inserted using ImageJ. **c** Individual colonies of MDA-pc3, MDA-B6 and MDA-B6-M cells were fixed and stained with crystal violet in 6-well plates. Scattered and compact colonies were counted in six wells per cell line, using an inverted microscope. The graph represents the percentage of scattered colonies in each cell line. **d** TNBC cells, BT-20, were transduced with EPHB6-targeting shRNAs (shB6-1 or shB6-2, individually), as indicated. Transduction with non-silencing shRNA (BT20-NS) was used as a control. EPHB6 expression was analysed as in **a**, and quantitated by densitometry. EPHB6 quantifications were normalised on matching tubulin controls and presented in arbitrary units (AU). **e** Formation of scattered and compact colonies by BT20-NS, BT20-shB6-1 and BT20-shB6-2 cells was analysed as in **c**. **f** Representative confocal microscopy images of BT20 cells with intrinsic EPHB6 expression (BT20) and of MDA-B6-M cells (MDA-B6-M) both co-stained with anti-EPHB6 (red) and anti-ZO-1 (green). Stainings with matching non-specific IgGs are shown as specificity controls. Images were captured using an LSM 700 Zeiss confocal microscope with 40× oil objective lens. Scale bar, 20 μm, was inserted using ZEN 2012 software. Red and green signal intensities were set using matching non-specific IgG controls as thresholds. Panels were generated using PowerPoint and Adobe Illustrator CS6 software. Data are shown as means ± SD. Experiments were performed at least three times; *P < 0.05; Student’s *t*-test

To address EPHB6 functions in TNBC, we used TNBC cells, MDA-MB-231, that are highly invasive, have passed epithelial-to-mesenchymal transition (EMT) [20] and lost EPHB6 expression [17, 18]. EPHB6 expression was restored by transfecting MDA-MB-231 with cDNAs encoding wild-type EPHB6 (MDA-B6) or Myc-tagged EPHB6 (MDA-B6-M) (Fig. 2a), while the empty pcDNA3 expression vector was used as a control (MDA-pc3), as we reported [15]. Our work with these cells revealed that EPHB6 efficiently suppresses their motility (Supplementary Figure S1B). When cultured in individual colonies, MDA-B6 and MDA-B6-M mostly formed very compact colonies with tight cell–cell contacts, while MDA-pc3 cells were predominantly organised in scattered colonies with loose cell–cell interactions (Fig. 2b, c). EPHB6 ability to reduce scattering was not limited to MDA-MB-231, as silencing EPHB6 expression in TNBC cells, BT-20, (Fig. 2d) that are innately EPHB6-positive (Supplementary Figure S1C), increased the formation of scattered colonies (Fig. 2e). Consistent with its apparent role in cell interactions, EPHB6 was frequently found in the areas of cell–cell contact formation in both MDA-B6-M and BT-20 cells and co-localised there with a tight junction protein, ZO-1 (Fig. 2f).

**Fig. 3:**
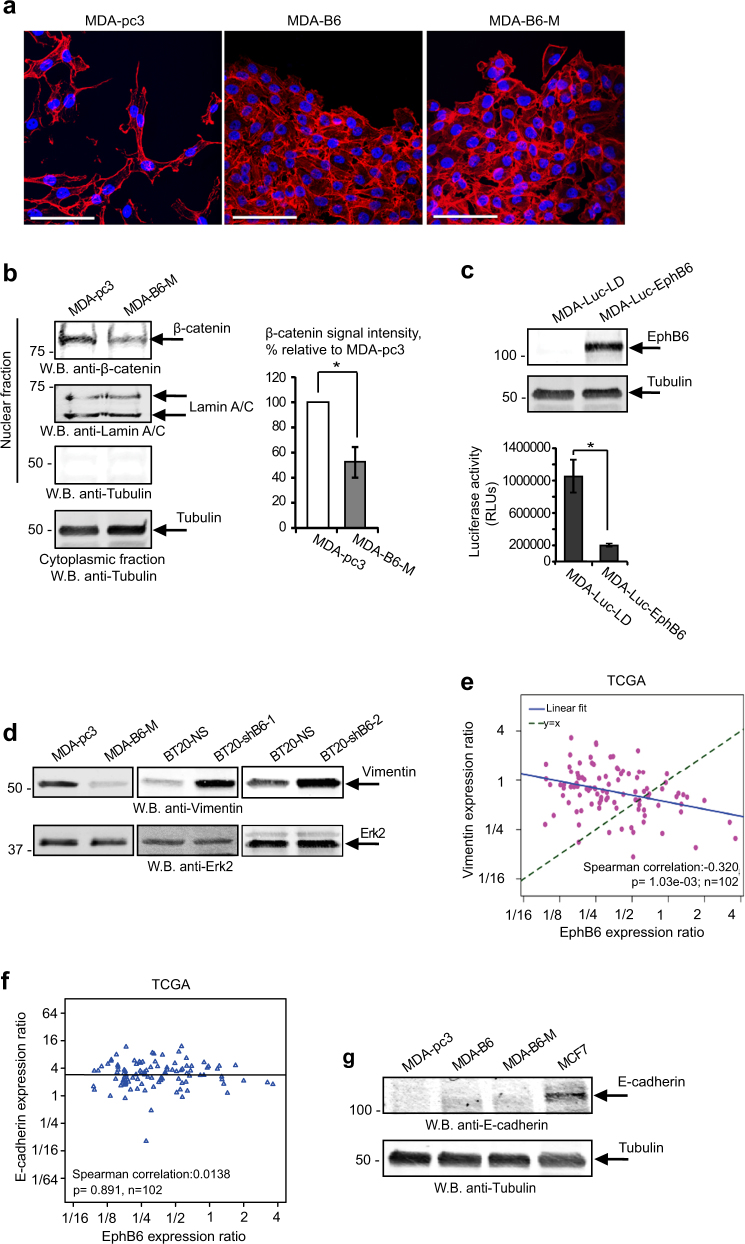
EPHB6 partially suppresses EMT. **a** Confocal microscopy images of representative areas of colonies formed by MDA-pc3, MDA-B6 and MDA-B6-M in Fig. 2b. Scale bar, 100 µm, inserted using ImageJ. **b** Nuclear fractions of MDA-pc3 and MDA-B6-M cells were prepared using the Nuclear/Cytosol Fractionation kit (BioVision), and β-catenin presence was examined by Western blotting. Purity of the nuclear fractions was evaluated by Western blotting with anti-tubulin. Equal aliquots of matching nuclear fractions were loaded separately and blotted with anti-lamin A/C. Results of Western blotting were quantitated by densitometry, β-catenin quantifications were normalised on matching lamin A/C signals and plotted as a percentage relative to MDA-pc3. The graph represents a summary of six independent experiments. **c** MDA-MB-231 were stably transduced with a lentiviral vector encoding a TCF/LEF luciferase reporter (MDA-Luc). MDA-Luc cells were transduced with an EPHB6-encoding vector (MDA-Luc-EphB6) or with an empty vector (MDA-Luc-LD) and EPHB6 expression in MDA-Luc-EPHB6 was confirmed by Western blotting. MDA-Luc-LD and MDA-Luc-EPHB6 cells were lysed, cell lysate samples containing 10 µg of total protein were mixed with the luciferase assay reagent and luciferase activity was measured with a luminometer. The graph represents a summary of four independent experiments. **d** Vimentin expression was assessed in the indicated cell lines by Western blotting. **e** Scatterplot representing EPHB6 and vimentin expression levels in TNBC samples from the TCGA dataset. Expression values are ratios as in Fig.1a. Included in the scatterplot is the Spearman correlation and the line showing the linear fit and identity. **f** Scatterplot representing EPHB6 and E-cadherin expression levels in TNBC samples from the TCGA dataset. Spearman correlation statistics and a linear fit are shown. **g** E-cadherin expression in the indicated cells was analysed by Western blotting. Breast cancer cells, MCF7, were used as a positive control. Data are shown as means ± SD. Experiments were performed at least three times; *P < 0.05; Mann–Whitney *U*-test (**b** and **c**)

As EMT is associated with increased cell motility and reduced contacts between cancer cells, our observations indicate that EPHB6 could antagonise this process. Indeed, EPHB6 restoration in MDA-MB-231 changed their morphology from irregular to cobblestone-like, typical for epithelial cells (Fig. 3a). This was accompanied by the reduced presence of β-catenin in the nuclei (Fig. 3b) and by the inhibition of transcription-enhancing β-catenin action, which actively supports EMT [21] (Fig. 3c). These findings correlated with a recent report, showing that EPHB6 reduces β-catenin expression [22]. To further analyse how EPHB6 impinges upon EMT, we evaluated its effect on the expression of an EMT marker, vimentin, which is usually present in mesenchymal cells and promotes this process [23]. Consistent with EMT-suppressing EPHB6 action, vimentin levels proved to be decreased in the presence of EPHB6, while EPHB6 silencing enhanced vimentin expression even in BT-20 cells (Fig. 3d) that had passed EMT [24].

Collectively, our data suggested an active role for EPHB6 in EMT suppression. In further support of this, our analysis of the TCGA database revealed that EPHB6 expression negatively correlates with that of vimentin in TNBC tumours (Fig. 3e). Interestingly, we did not detect any correlation between EPHB6 and an epithelial marker, E-cadherin, in the TCGA dataset and no significant effect of EPHB6 on E-cadherin expression was evident in our experiments (Fig. 3f, g). These observations indicate that although EPHB6 consistently inhibits EMT-associated behaviour of cancer cells and shifts the balance in favour of the mesenchymal-to-epithelial transition (MET), it achieves only partial EMT suppression. This was further supported by our findings, showing that although EPHB6 does not produce consistent effects on EMT/MET markers, the Met receptor and laminin, it significantly decreases the expression of an EMT-related protein, N-cadherin, in TNBC cells (Supplementary Figure S1D).

### The EPHB6 receptor increases tumour-initiating activity

**Fig. 4:**
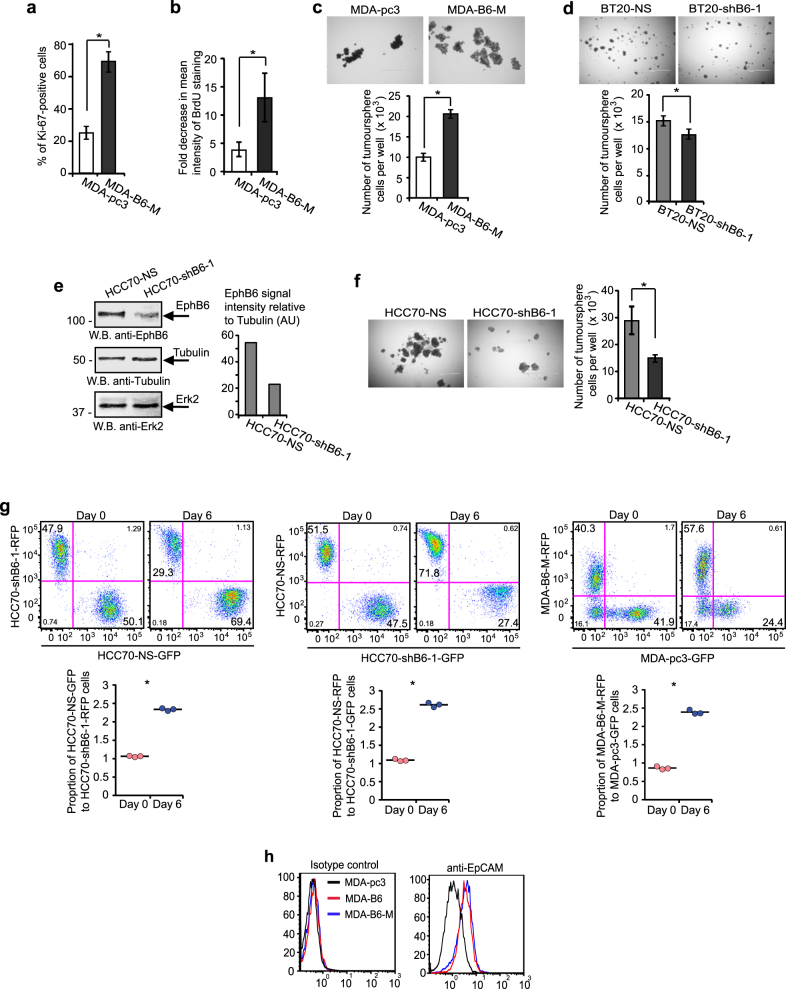
EPHB6 increases proliferation of tumoursphere-producing cells. **a** Cells were seeded into Ultra-Low Attachment 24-well plates (4 × 10^3^ cells per well) to form tumourspheres for 7 days. Tumourspheres were dissociated and analysed by flow cytometry for Ki-67 in duplicates. Gating was based on matching isotype control. The graph represents analysis of three independent experiments and shows percentage of Ki-67-positive cells in each cell line. **b** Cells were labelled with 10 µM BrdU for 48 h in monolayers using BrdU kit (R&D Systems). Initial levels of BrdU incorporation were examined by flow cytometry. Remaining cells were seeded in the BrdU-free medium to proliferate in tumourspheres for 7 days, as in **a**. Tumourspheres were dissociated and BrdU loss was assessed by flow cytometry. The graph represents the analysis of three independent experiments and shows fold decrease in mean intensity of BrdU staining in each cell line relative to matching initial measurements. **c** To directly assess the rate of proliferation of tumoursphere-forming cells, MDA-pc3 or MDA-B6-M cells were seeded into 24-well Ultra-Low attachment plates (4 × 10^3^ cells per well) and allowed to propagate in tumourspheres for 7 days. Representative tumourspheres were imaged. For each replicate, tumourspheres from 12 independent wells were pulled together, dissociated into a single-cell suspension, counted and the average number of cells per well was calculated. The graph represents the analysis of three independent experiments performed in duplicates. **d** The indicated cells were propagated in tumourspheres and analysed as in **c**. **e** TNBC cells, HCC70, were transduced with non-silencing shRNA (HCC70-NS) or EPHB6-targeting shRNA, shB6-1, and EPHB6 expression was analysed as in Fig. 2d. **f** The indicated cells were propagated in tumourspheres and analysed as in **c**. **g** GFP- or RFP-expressing HCC70-NS cells (HCC70-NS-GFP or HCC70-NS-RFP) were mixed in equal numbers (1:1 ratio) with RFP- or GFP-expressing HCC70-shB6-1 cells (HCC70-shB6-1-RFP or HCC70-shB6-1-GFP), as indicated and allowed to proliferate in tumourspheres for 6 days. Samples of mixed cells were collected at day 0 and day 6, and analysed by flow cytometry and the FlowJo software in triplicates. In addition, RFP-expressing MDA-B6-M (MDA-B6-M-RFP) cells mixed in equal numbers (1:1 ratio) with GFP-expressing MDA-pc3 (MDA-pc3-GFP), cells were propagated and analysed as described above for HCC70. Each graph represents analysis of triplicates and shows the proportion of GFP- and RFP-expressing cells, as indicated for each combination, at seeding (Day 0) and after 6 days of propagation in tumourspheres. Solid lines in the graph indicate mean values. **h** EpCAM expression was analysed by flow cytometry. Data are shown as means ± SD. Experiments were performed at least three times; *P < 0.05; Student’s *t*-test or Mann–Whitney *U*-test. Scale bars, 1000 µM. For optimal presentation, individual tumoursphere images are shown at different brightness and contrast settings

**Fig. 5:**
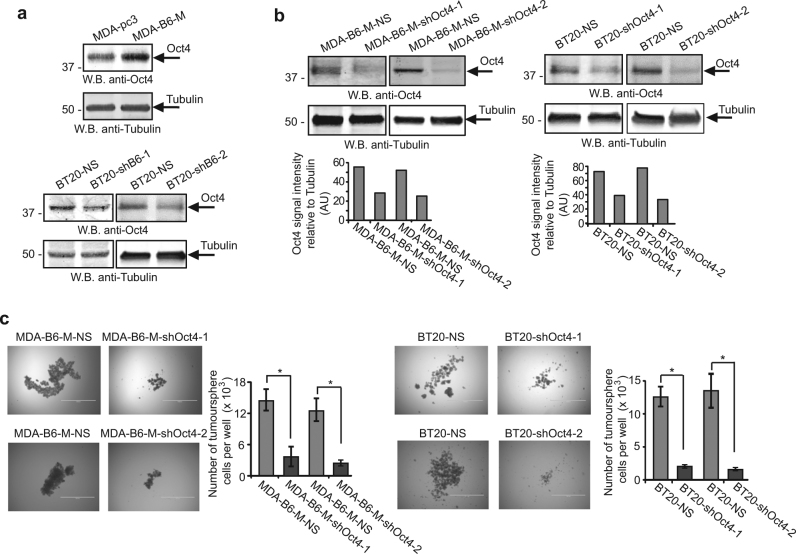
EPHB6 relies on elevated OCT4 expression to enhance proliferation of tumoursphere cells. **a** Total cell lysates were prepared from the indicated cells using Lysis Buffer from the Proteome Profiler Stem Cell Array Kit (R&D) and OCT4 expression was assessed by Western blotting. **b** MDA-B6-M and BT-20 cells were stably transduced with non-silencing shRNA or OCT4-targeting shRNAs (shOct4-1 or shOct4-2, individually), as indicated. OCT4 expression was assessed by Western blotting with anti-OCT4 and quantitated as in Fig. 2d. **c** The indicated cells were seeded into 96-well Ultra-Low attachment plates (2 × 10^3^ cells per well), allowed to propagate in tumourspheres for 7 days and analysed as in Fig. 4c. Graphs represent analyses of three independent experiments. Data are shown as means ± SD. Experiments were performed at least three times; *P < 0.05; Mann–Whitney *U*-test. Scale bars, 1000 µM. For optimal presentation, individual tumoursphere images are shown at different brightness and contrast settings

Strong evidence has been presented to support both a model, where EMT favours tumour initiation [25], and a notion, suggesting that MET promotes TICs generation [26]. Therefore, we examined EPHB6 effect on proliferation of TNBC cells producing tumourspheres, as tumourspheres better represent tumour behaviour than cells cultured in monolayers and are predominantly formed by TICs [27–34]. Analyses of Ki-67 staining and BrdU retention showed that EPHB6 increases cell proliferation in these structures (Fig. 4a, b). The biological relevance of this effect was confirmed by our findings that EPHB6 expression significantly accelerates expansion of tumoursphere cells (Fig. 4c–g, Supplementary Figure S1E and F). Although the effect of EPHB6 silencing was relatively limited in BT-20 in comparison to the effect observed in TNBC cells, HCC70 (Fig. 4d, f) that also express EPHB6 (Supplementary Figure S1C), it was statistically significant and consistently observed (Fig. 4d, Supplementary Figure S1E and F). Overall, these observations indicated that EPHB6 could be involved in the regulation of TIC propagation. Indeed, EPHB6 presence strongly enhanced expression of EpCAM (Fig. 4h), which was previously characterised as breast cancer TIC marker [29, 35]. Moreover, EPHB6 also elevated expression of the OCT4 transcription factor (Fig. 5a) that supports TIC activity [36, 37]. Consistent with its reported function in breast cancer TICs [36, 37], OCT4 silencing strongly reduced the expansion of tumourspheres (Fig. 5b, c), while not decreasing EPHB6 expression (Supplementary Figure S1G). Taken together, this suggested that EPHB6 ability to augment expansion of tumoursphere cells depends on the elevated OCT4 expression.

**Fig. 6:**
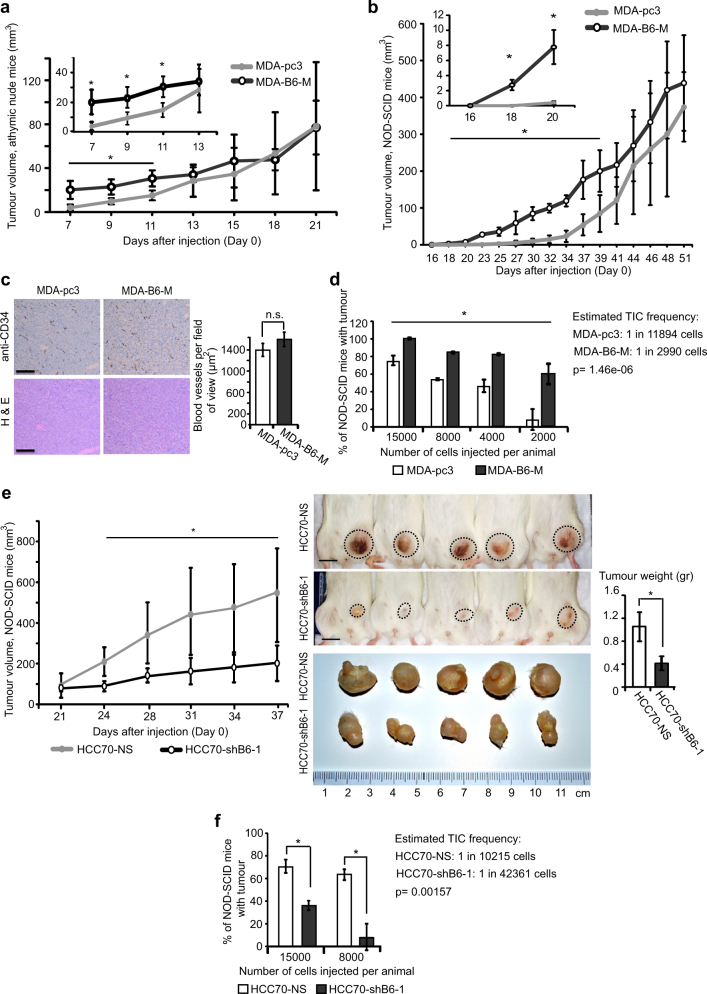
EPHB6 accelerates tumour growth and supports tumour initiation. **a**, **b** MDA-pc3 and MDA-B6-M cells were inoculated into the mammary fat pad region of 4–6-week-old female athymic nude mice (*n* = 7 per group; 1.5 × 10^6^ cells per mouse) (**a**) or NOD-SCID mice (*n* = 7 per group; 1.5 × 10^5^ cells per mouse) (**b**), and tumour growth was monitored, as indicated. Tumour volume was calculated by the equation: *A*/2×*B*^2^, where *A* was long and *B* was short diameter of the tumour. **c** MDA-pc3 and MDA-B6-M were injected into NOD-SCID mice as in **b** (*n* = 4 per group). Tumours were processed for staining with anti-CD34 or haematoxylin and eosin (H&E) staining. For immunohistochemistry, tumours were fixed in 10% formalin, paraffin embedded and 4-µm-thick sections were affixed on slides. Dewaxing and antigen retrieval were performed using the Dako PT Link system (Dako Canada Inc., Mississauga, ON, Canada). Staining was performed on Dako Autostainer Link using anti-CD34 and the Dako FLEX DAB+ Detection Kit. In each section, 12, 3, 6 and 9 o’clock fields were imaged with a Nikon Eclipse E400 microscope (Nikon Instruments Inc., Melville, NY, USA) at 100× magnification and blood vessel density was quantified using the ImageJ software. The graph summarises two independent experiments. Scale bar, 100 µm. **d** Decreasing numbers of MDA-pc3 or MDA-B6-M were injected into NOD-SCID mice and tumour formation was monitored for 60 days. The graph summarises two independent experiments. At least five animals per condition were used in each experiment. **e** HCC70-NS and HCC70-shB6-1 cells were injected into NOD-SCID mice (*n* = 5 per group; 1.5 × 10^6^ cells per mouse) and tumour growth was monitored. Upon experiment termination, mice were photographed; scale bar, 10 mm. Tumours were photographed and weighed. **f** Tumour formation by decreasing numbers of HCC70-NS or HCC70-shB6-1 in NOD-SCID mice was analysed as in **d**. The graph summarises data from two independent experiments. At least five animals per condition were used in each experiment. Data are shown as means ± SD. Animals were randomly assigned to all experimental groups. Experiments were performed at least two times. TIC frequencies were estimated using the Extreme Limiting Dilution Analysis [63]; *P < 0.05; Student’s *t*-test or Mann–Whitney *U*-test; n.s. statistically not significant

Since TICs are responsible for tumour initiation and self-renewal, their increased proliferation should result in a higher rate of initial tumour growth. To assess EPHB6 effect on TNBC tumours, we injected MDA-pc3 or MDA-B6-M cells into mammary fat pads of NU(NCr)-Foxn1^nu^ (athymic) and NOD.Cg-Prkdc^scid^ Il2rg^tm1Wjl^/SzJ (NOD-SCID) mice (1.5 × 10^6^ cells and 1.5 × 10^5^ cells per animal, respectively). In both models, mice developed tumours with higher initial growth rates, when injected with EPHB6-expressing cells (Fig. 6a, b). Staining for a blood vessel marker, CD34, showed no difference in vascularisation levels (Fig. 6c), indicating that EPHB6 ability to enhance tumour growth was not because of its effect on neovascularization.

To assess EPHB6 effect on tumour initiation, we injected mice with decreasing doses of cancer cells. This consistently resulted in more frequent development of EPHB6-positive tumours, and statistical analysis confirmed that EPHB6 supports expansion of TIC populations (Fig. 6d). To further validate these observations, we challenged NOD-SCID mice with TNBC cells, HCC70. Consistent with our initial results, silencing of EPHB6 expression in HCC70 decreased tumour growth and tumour initiation, and reduced TIC frequency (Fig. 6e, f). Western blot analyses confirmed that introduced alterations in EPHB6 expression were preserved in both MDA-MB-231 and HCC70 xenograft models (Supplementary Figure S1H). In addition, a similar effect of EPHB6 silencing on tumour growth was observed with TNBC cells, BT-20 (Supplementary Figure S2A). Collectively, these data strongly support the notion that EPHB6 enhances expansion of TIC populations and promotes tumour development. Interestingly, we did not observe any consistent effect of EPHB6 on the CD24^lo^CD44^hi^ combination, identifying some breast cancer TIC populations and the ALDEFLUOR assay has not revealed a consistent effect on another TIC marker, ALDH1. This lack of effect on some TIC markers indicated that in the analysed cell lines, EPHB6 enhanced tumour initiation by expanding a restricted subset of TICs that expressed higher levels of OCT4 and accelerated tumour growth.

### EPHB6 effect on TNBC cells is mediated by the Ras-Erk pathway

**Fig. 7:**
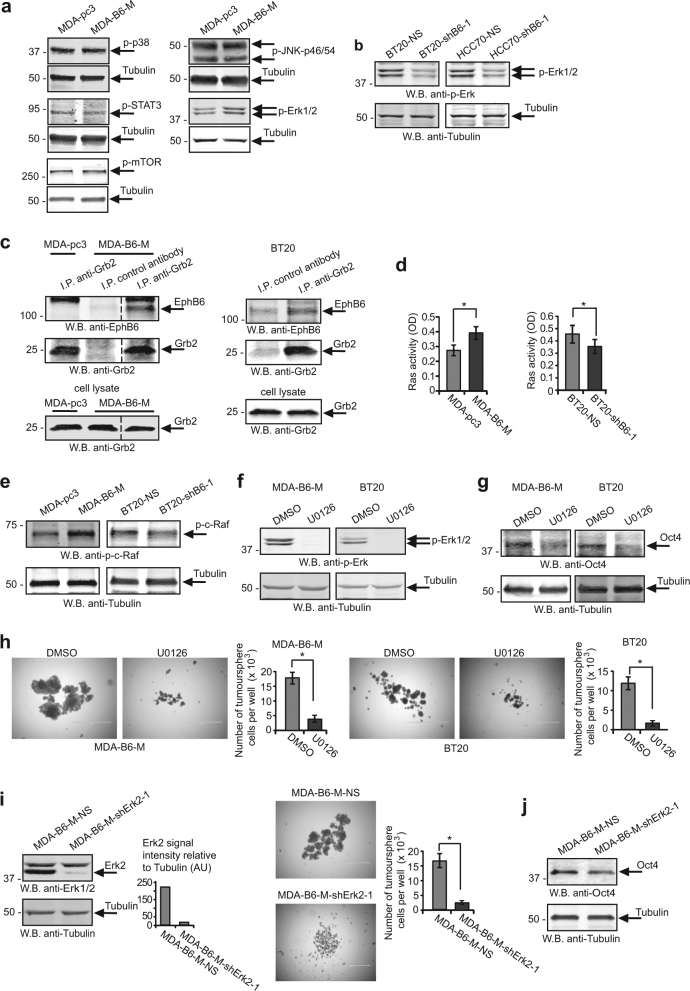
EPHB6 relies on the ERK pathway for executing its responses in tumourspheres. **a** Phosphorylation status of the indicated proteins was examined by Western blotting with phospho-specific antibodies. Blotting or re-blotting of the same membranes with anti-tubulin was used as a loading control. For anti-p-JNK analysis, equal aliquots of matching cell lysates were loaded twice on the same gel and blotted with either anti-phospho-JNK or anti-tubulin, since p-JNK and tubulin signals overlapped. **b** Phosphorylation of ERK kinases was assessed by Western blotting. **c** The indicated cells were lysed and immunoprecipitations were performed with anti-GRB2 or a matching amount of non-specific control antibody. Immunoprecipitates were resolved by non-reducing SDS PAGE and EPHB6 presence was analysed by Western blotting. Presence of GRB2 in immunoprecipitates or input lysates was monitored by Western blotting. Removed irrelevant lanes are indicated by dashed lines. **d** The indicated cells were serum starved for 24 h and analysed for Ras activation using the G-LISA Ras Activation Assay Biochem Kit (Cytoskeleton Inc). Ras activation is presented in optical density units (OD). Graphs represent analyses of three independent experiments. **e** Cells were analysed by Western blotting for activating c-Raf phosphorylation at Ser338. **f** Cells were treated with U0126 (10 μM) or DMSO at 37 °C for 2 h and phosphorylation status of ERK kinases was assessed by Western blotting. **g** MDA-B6-M and BT-20 cells were cultured for 72 h with U0126 (10 μM) or a matching volume of DMSO, and OCT4 expression was assessed by Western blotting. **h** Proliferation of tumoursphere cells in the presence of U0126 (10 μM) was examined as in Fig. 5c. **i** MDA-B6-M cells were transduced with non-silencing shRNA (MDA-B6-M-NS) or ERK2-targeting shRNA, shErk2-1. ERK2 expression was assessed as in Fig. 2d. The effect of ERK2 silencing on cell proliferation in tumourspheres was analysed as in Fig. 5c. **j** OCT4 expression was monitored by Western blotting. Data are shown as means ± SD. Experiments were performed at least three times; *P < 0.05; Student’s *t*-test or Mann–Whitney *U*-test. Scale bars, 1000 µM. For optimal presentation, individual tumoursphere images are shown at different brightness and contrast settings

To gain an understanding of cytoplasmic signalling used by EPHB6 to augment cell proliferation in tumourspheres, we monitored its effect on essential signalling pathways. While we could not detect any consistent effect of EPHB6 on p38, STAT3, mTOR or JNK, EPHB6 enhanced the activating phosphorylation of the ERK1 and ERK2 kinases (Fig. 7a, b, Supplementary Figure S3A). As RTKs typically interact with the GRB2 adaptor protein to activate the RAS-RAF-MEK-ERK pathway [38], we examined if EPHB6 is also associated with GRB2. Indeed, we consistently observed EPHB6 in GRB2 immunoprecipitates (Fig. 7c). Moreover, EPHB6 expression triggered the activation of the RAS GTPase and RAF kinase (Fig. 7d, e), and EPHB6-induced Erk phosphorylation relied on MEK activity (Fig. 7f), indicating that EPHB6 uses the conventional RAS-MAPK cascade for activating ERK kinases. ERK kinases are known to enhance OCT4 expression [39], and to promote pluripotency in human cells [39, 40] and therefore could be essential for mediating EPHB6 responses. A crucial role for ERK signalling was confirmed in our experiments, showing that blockage of ERK activation with MEK inhibitors negated EPHB6 effects, reducing OCT4 expression and curbing cell proliferation in tumourspheres (Fig. 7g, h; Supplementary Figure S3B–D). In agreement, ERK2 silencing strongly inhibited EPHB6-triggered proliferative responses and OCT4 expression (Fig. 7i, j; Supplementary Figure S3E–G).

### The EPHB6 receptor reduces resistance of TNBC tumours

**Fig. 8:**
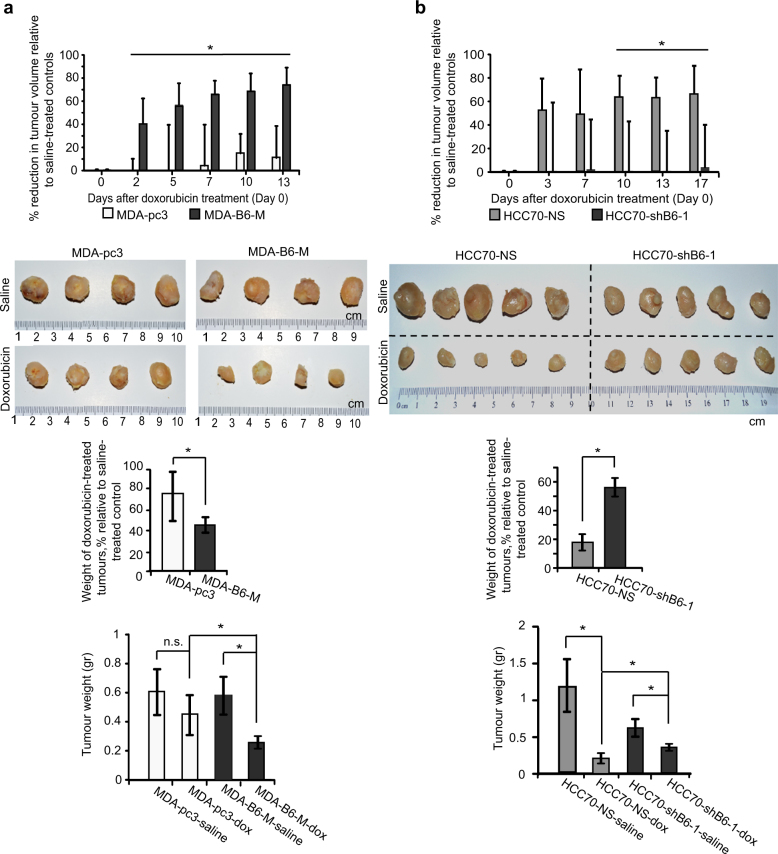
EPHB6 reduces tumour drug resistance. **a** MDA-pc3 and MDA-B6-M were injected into the mammary fat pad of NOD-SCID mice (1.5 × 10^6^ cells per mouse). Mice with tumours were treated weekly with i.v. injections of doxorubicin (2 mg/kg) or saline (*n* = 5 per group). Day 0 indicates the day of the initial treatment. The reduction in tumour growth in doxorubicin-treated mice is presented as a percentage relative to matching saline-treated controls. Upon experiment termination, tumours were photographed and weighed. Only four animals per group were included in these final measurements, due to earlier tumour-unrelated lethality. The middle graph represents tumour weights in doxorubicin-treated mice as a percentage relative to matching saline-treated controls. Average tumour weights for each group of experimental animals are shown in the lower graph. **b** HCC70-NS and HCC70-shB6-1 were injected into NOD-SCID mice and effectiveness of doxorubicin treatment was assessed as in **a**. Data are shown as means ± SD. Animals were randomly assigned to all experimental groups. Experiments were performed at least two times; *P < 0.05; Student’s *t*-test or Mann–Whitney *U*-test; n.s. statistically not significant

Increased tumour-initiating activity is expected to result in enhanced drug resistance [41, 42]. To examine if EPHB6 supports cancer drug resistance, we treated mice with MDA-pc3 or MDA-B6-M tumours with a DNA-damaging drug, doxorubicin, which is frequently used in TNBC therapy. To our surprise, growth of MDA-B6-M tumours was strongly inhibited by doxorubicin, while it produced a very small effect on MDA-pc3 tumours (Fig. 8a). Moreover, doxorubicin also efficiently suppressed tumours initiated by HCC70 cells, while EPHB6 silencing enhanced their resistance (Fig. 8b).

**Fig. 9:**
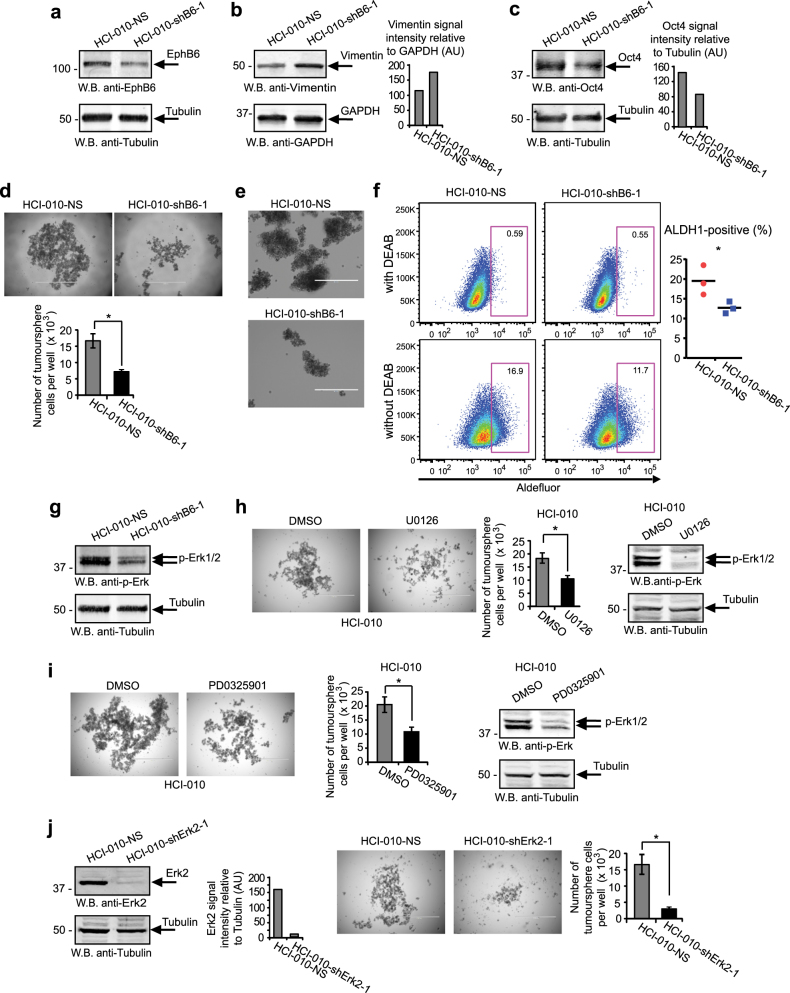
EPHB6 controls the behaviour of TNBC patient-derived xenograft. **a**–**d** Cells isolated from a TNBC patient-derived xenograft, HCI-010, were transduced with non-silencing shRNA (HCI-010-NS) or EPHB6-targeting shRNA (shB6-1) and analysed by Western blotting for EPHB6 (**a**), vimentin (**b**) and OCT4 (**c**) expression; proliferation of these cells in tumourspheres was analysed as in Fig. 5c, scale bar, 1000 µM (**d**). **e** Representative images of HCI-010-NS and HCI-010-shB6-1 tumourspheres shown at a higher magnification; scale bar, 400 µM. **f** HCI-010-NS and HCI-010-shB6-1 cells were analysed by flow cytometry to determine the proportion of aldehyde dehydrogenase 1 (ALDH1)-positive cells using the ALDEFLUOR assay kit (Stemcell Technologies). The assay was performed according to the manufacturer’s instructions. Representative flow cytometry profiles including the negative controls (cells treated with DEAB, a specific ALDH1 inhibitor) are shown. The graph represents the analysis of triplicates and shows the proportion of ALDH1-positive cells in each cell line after subtracting the background values obtained from the matching negative controls. Solid lines in the graph indicate mean values. The data represent one of two independent experiments. **g** ERK phosphorylation was monitored by Western blotting. **h** Proliferation of HCI-010 cells in tumourspheres in the presence of U0126 (10 μM) was analysed as in Fig. 5c; scale bar, 1000 µM. Effect of U0126 on ERK phosphorylation was confirmed by Western blotting. **i** Proliferation of HCI-010 cells in tumourspheres in the presence of PD0325901 (100 nM) was analysed as in Fig. 5c; scale bar, 1000 µM. Effect of PD0325901 on ERK phosphorylation was confirmed by Western blotting. **j** HCI-010 cells were transduced with ERK2-targeting shRNA (shErk2-1). ERK2 silencing was confirmed as in Fig. 2d. Proliferation of cells in tumourspheres was analysed as in Fig. 5c; scale bar, 1000 µM. Data are shown as means ± SD. Experiments were performed at least three times until otherwise indicated; *P < 0.05; Student’s *t*-test or Mann–Whitney *U*-test. For optimal presentation, individual tumoursphere images are shown at different brightness and contrast settings

**Fig. 10:**
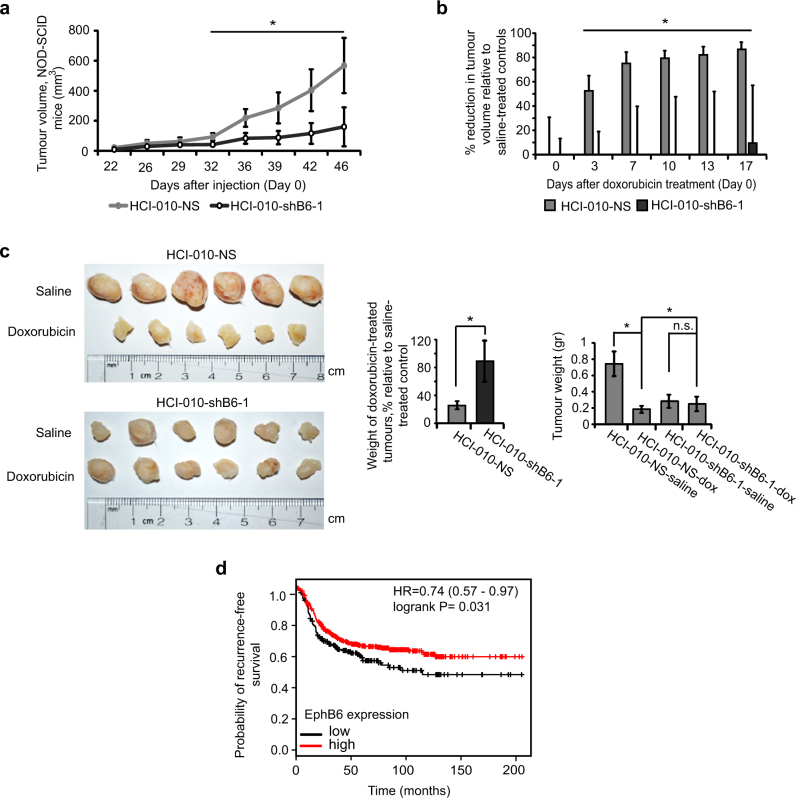
EPHB6 enhances growth of TNBC tumours and improves their drug sensitivity. **a**–**c** HCI-010-NS and HCI-010-shB6-1 cells were injected into the mammary fat pad region of 4–6-week-old NOD-SCID mice (1 × 10^6^ cells per mouse). Mice with tumours were treated with doxorubicin or saline (*n* = 6 per condition) as in Fig. 8a. Growth rates of saline-treated HCI-010-NS and HCI-010-shB6-1 tumours were compared (**a**). Doxorubicin-induced reductions in growth of HCI-010-NS and HCI-010-shB6-1 tumours are presented as a percentage relative to matching saline-treated controls (**b**). Upon termination of the experiment at day 46 following initial cell injections, the excised tumours were photographed and weighed (**c**). Tumour images from one of two independent experiments are presented. The left graph in **c** represents tumour weights in doxorubicin-treated mice as a percentage relative to matching saline-treated controls. Average tumour weights for each group of experimental animals are shown in the right graph. The graphs represent analysis of two independent experiments. **d** Kaplan–Meier curve showing the probability of recurrence-free survival in 581 patients with basal breast cancer. The lower tertile of EPHB6 expression level was used as a cutoff. The 95% confidence interval for the hazard ratio (HR) is shown in parentheses. Probabilities for patients with EPHB6 levels in their tumours above the lower tertile are shown in red, while the probability for those with EPHB6 levels below the lower tertile are shown in black. Data are shown as means ± SD. Animals were randomly assigned to all experimental groups. Experiments were performed at least two times; *P < 0.05; Student’s *t*-test or Mann–Whitney *U*-test; n.s. statistically not significant

The relevance of our findings was further supported by our work with a TNBC patient-derived xenograft (PDX) model, HCI-010, which closely follows tumour behaviour [43]. HCI-010 tumours are EPHB6-positive and there, EPHB6 action mimicked responses that we initially observed in TNBC cell lines. EPHB6 silencing in HCI-010 increased the vimentin level, suppressed OCT4 expression and inhibited expansion of tumoursphere cells (Fig. 9a–e, Supplementary Figure S4A–D). Interestingly, consistent with EPHB6 ability to support tumoursphere expansion and tumour initiation (Fig. 6d, f), its silencing significantly suppressed ALDH1-positive HCI-010 population, which is expected to maintain TIC activity [44] (Fig. 9f, Supplementary Figure S4E). Reduced EPHB6 expression also decreased ERK activation (Fig. 9g, Supplementary Figure S4F), and cell proliferation in tumourspheres proved to depend on EPHB6-activated ERK signalling (Fig. 9h–j, Supplementary Figure S4G). In NOD-SCID mice, PDX HCI-010 tumours were treated with doxorubicin or saline, as a control. Consistent with EPHB6 action in TNBC cell lines, EPHB6 silencing reduced growth rates in saline-treated tumours, while increasing tumour resistance to doxorubicin (Fig. 10a–c, S5A–C). As in cell lines-based models, EPHB6 silencing was maintained in PDX tumours (Supplementary Figure S4H).

Dormant slow-proliferating TICs are likely to escape cytotoxic therapies targeting fast-proliferating cells and are responsible for cancer relapse [42, 45, 46]. Taken together, our observations suggest an intriguing model, whereby EPHB6 accelerates expansion of TNBC TICs, while also increasing tumour sensitivity to DNA-damaging compounds and potentially other therapies selectively affecting proliferating cells, probably by driving TICs out of their slow-proliferating, resistant state. This model is further supported by our finding that EPHB6 increases killing of tumoursphere cells by therapeutic compounds that depend on high rates of cell proliferation, such as doxorubicin or docetaxel, while not improving elimination of cancer cells in monolayers, where EPHB6 has no significant effect on cell propagation [47] (Supplementary Figure S6A and B). Moreover, EPHB6 does not increase the sensitivity of tumoursphere cells to bortezomib, a cancer drug that acts as a proteasomal inhibitor and should not directly depend on the proliferative activity (Supplementary Figure S6C). Since TNBC is mostly treated with compounds acting on fast-propagating cells, including doxorubicin and taxane derivatives [48, 49], our model predicts better relapse-free survival of TNBC patients with high EPHB6 expression in their tumours. Consistent with this, our analysis revealed a positive correlation between EPHB6 expression and the long-term recurrence-free survival of breast cancer patients with basal-like tumours that mostly represent TNBC [50] (Fig. 10d).

## Discussion

TNBC tumours represent the most lethal type of breast cancer because of the high level of drug resistance, high metastasis and lack of targeted therapies [16]. An understanding of mechanisms governing TNBC cells is critical for the development of efficient treatments for this aggressive malignancy. Our findings show that in agreement with its previously reported anti-invasive properties [15, 47], EPHB6 partially reverses the EMT phenotype in TNBC cells. Our observations also indicate that partial EMT suppression induced by EPHB6 is associated with the expansion of tumoursphere cell populations that are mostly represented by TICs [27–34]. Moreover, EPHB6 action also results in the elevated OCT4 expression and in the enlarged ALDH1-positive population in the HCI-010 PDX. Since these factors are characteristic traits of TICs [29, 35–37, 44, 51], our data strongly support a model, whereby the EPHB6 receptor concurrently suppresses EMT and promotes proliferation of TNBC TICs. In agreement, EPHB6 augments tumour initiation and expansion of TIC populations in animal models, ultimately confirming the role for this receptor in TIC biology. Nevertheless in established cell lines, MDA-MB-231 and BT-20, EPHB6 presence did not expand CD24^lo^CD44^hi^ or ALDH1-positive subpopulations, selectively increasing EpCAM expresion that represents some breast cancer TICs. This suggests that EPHB6 does not have a blanket effect on TICs, but most likely promotes specific subsets of these cells, and agrees very well with TIC heterogeneity, which results from the evolution of TICs in developing tumours [52, 53]. Our observations also reflect an unfortunate reality that there is no universal marker that defines all types of breast cancer TICs [51, 54] and indirectly suggest that EPHB6 itself may serve as a TIC marker in TNBC.

**Fig. 11:**
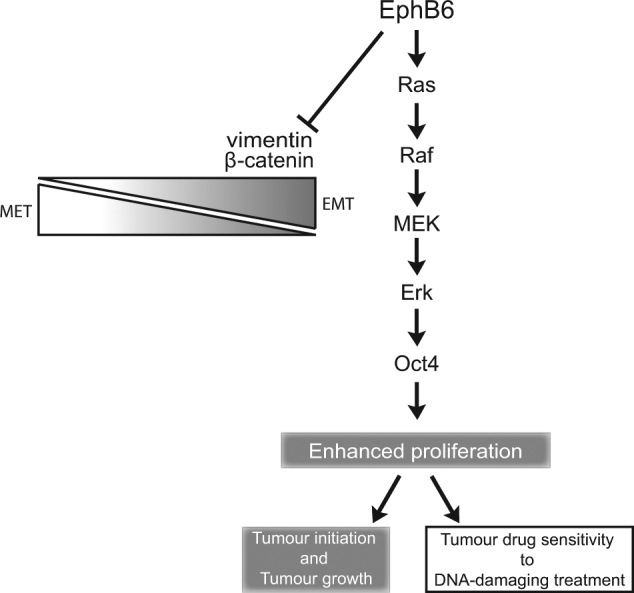
A schematic presentation of EPHB6 action in TNBC

OCT4 is known to support cell proliferation [55] and EPHB6-induced proliferation in tumourspheres proved to rely on the increased OCT4 expression. This represents a novel mechanism of the regulation of cell proliferation, as Eph receptors have not been shown to control OCT4 activity. EPHB6 has been previously reported by our team and other groups to activate ERK kinases; however, the relevance of this signalling to cell proliferation and its mechanism have not been addressed [22, 56, 57]. Our investigation reveals that EPHB6 interacts with GRB2 and uses RAS-ERK signalling to elevate OCT4 expression. In agreement, inhibition of the Erk pathway also blocks EPHB6 ability to enhance cell proliferation in tumourspheres. Taken together, our observations support a model, where the EPHB6 receptor acts in TNBC to partially reverse EMT, while its signalling through the RAS-ERK cascade increases OCT4 expression, thus augmenting expansion of TIC populations (Fig. 11). This agrees with a report, demonstrating that the epithelial-like state is associated with a high proliferative activity in breast cancer TICs [58]. This is also consistent with previous observations, showing that ERK2 enhances TIC-like characteristics in immortalised breast epithelial cells [59].

EPHB6 activity in supporting cell proliferation suggests that it should be beneficial for some TNBC tumours to maintain its expression. Indeed, analyses of the TCGA and EBI databases reveal that although EPHB6 expression is generally reduced in breast cancer, it is better preserved in TNBC tumours. While some of these results differ from the previous report, suggesting that EPHB6 expression may be increased in overall breast cancer [60], this difference is likely a reflection of the smaller database used in the earlier investigation.

Remarkably, tumours formed by EPHB6-expressing TICs proved to be much more sensitive to treatment with doxorubicin, which could be in part due the ability of EPHB6 to convert TICs into faster proliferating, less resistant cells. Targeting TICs is an important approach in cancer therapy [29, 46, 61] and counterintuitively, our results caution against inhibiting molecules that support TIC proliferation, when using DNA-damaging drugs, as this is likely to drive TICs into a drug-resistant state. Our investigation also indicates that it could be beneficial to support EPHB6 activity in TNBC tumours, when using conventional DNA-damaging treatment, as this would improve tumour elimination, while also suppressing invasive properties of cancer cells.

Taken together, our observations reveal a new molecular mechanism, whereby the EPHB6 receptor controls initiation, growth and drug sensitivity of TNBC tumours, and are likely to have a broad impact on the development of effective therapeutic strategies for this aggressive malignancy.

## Materials and methods

### Antibodies

Anti-β-tubulin (sc-9104), anti-GRB2 (sc-255), anti-β-catenin (sc-7199), anti-EPHB6 (sc-134332), anti-ERK1/2 (sc-94), anti-ERK2 (sc-154) and anti-Met (sc-10) were from Santa Cruz, anti-EPHB6 (SAB1403784) was from Sigma. Anti-OCT4 was from Cell Signaling Technology (2840) or STEMCELL Technologies (60093). Anti-CD34 (ab81289) and pan-specific anti-laminin (ab7463) were from Abcam. Anti-E-cadherin (610182) and anti-Ki-67 (550609) were from BD Biosciences. Anti-phospho-mTOR (AF1665) was from R&D Systems. Anti-phospho-Erk1/2 (4370), anti-phospho-p38 (4511), anti-phospho-STAT3 (9145), anti-phospho-JNK (4668), anti-EpCAM (2929), anti-GAPDH (2118) and anti-N-cadherin (4061) were from Cell Signaling. Anti-phospho-c-Raf (05–538) was from Millipore.

### Cell culture

MDA-MB-231, BT-20 and HCC70 were obtained from ATCC and passaged for less than three months following resuscitations. Therefore, no additional authentication was performed. Mycoplasma testing was performed. For culturing in individual colonies, cells were seeded into 6-well plates (1 × 10^3^ cells per well) and grown for 5–7 days. HCI-010 were previously characterised by one of the authors [43]. Single-cell preparations were made from HCI-010 tumours, as described [62]. HCI-010 cells were cultured in Ultra-Low attachment plates (Corning) using DMEM/F-12 medium, containing B27 supplement (1× Gibco), gentamicin (50 µg/ml; Gibco), hEGF (20 ng/ml; BPS Bioscience), bFGF (20 ng/ml; StemRD), insulin (10 µg/ml, Gibco), hydrocortisone (0.5 µg/ml; STEMCELL) and heparin (2 µg/ml; STEMCELL).

### Flow cytometry

Cells were fixed in 3% paraformaldehyde or 70% ethanol, incubated with indicated antibodies for 40–60 min, rinsed twice (0.5% BSA in PBS), stained with secondary antibodies, and analysed using Beckman Coulter Epics XL (Beckman Coulter Canada, LP., Mississauga, ON, Canada) or Miltenyi Biotec MACSQuant VYB (Miltenyi Biotec Inc. Auburn, CA, USA) flow cytometers and FlowJo software.

### Proliferation in tumourspheres

Cells were seeded into 24- or 96-well Ultra-Low attachment plates (4 × 10^3^ or 2 × 10^3^ cells per well, respectively) in complete Mammocult medium (STEMCELL) and allowed to propagate in tumourspheres for 7 days. For each replicate, tumourspheres from 12 independent wells were combined and dissociated using Trypsin-EDTA (Gibco), and proliferation was assessed by cell counting. Images were obtained using EVOS FL Cell Imaging System microscope (Life Technologies).

### Xenograft models

All protocols were approved by the University of Saskatchewan Animal Research Ethics Board (AREB). Female athymic nude mice (4–6 weeks old) were from Charles River Laboratories. Breeder pairs of NOD SCID mice were from The Jackson Laboratory. Cells were injected in mammary fat pad regions of 4–6-week-old female mice in 100 µl PBS. Tumours were measured using a digital caliper, tumour volume was calculated as: *A*/2×*B*^2^ (*A* and *B* are the long and short tumour diameters, respectively). For doxorubicin treatment, all experimental groups initially had similar average tumour sizes, animals were randomly assigned in all other experiments. Experiments were performed in a non-blinded fashion and terminated according to AREB guidelines.

TICs frequencies were estimated using the Extreme Limiting Dilution Analysis (http://bioinf.wehi.edu.au), as described [63].

### Luciferase reporter assay

MDA-MB-231 were transduced with Cignal Lenti TCF/LEF Luciferase lentiviral particles (Qiagen) (MDA-Luc) and selected with puromycin (10 µg/ml).

Human EPHB6 cDNA was amplified by PCR using the Elongase Enzyme Mix (Life Technologies) and EPHB6-specific primers: 5′-GGGGACAAGTTTGTACAAAAAA

GCAGGCTTCGCGGGCATGGTGTGTAGCCTATGG-3′, and 5′-GGGGACCACTTTGTA

CAAGAAAGCTGGGTCTCAGACCTCCACTGAGCC-3′. PCR products were inserted into attP1 and attP2 sites of the pDONR221 vector using Gateway BP Clonase Enzyme Mix (Life Technologies) to generate the EPHB6 entry clone. EPHB6 cDNA from the entry clone was transferred into attR1 and attR2 sites of the pLenti CMV Hygo DEST plasmid (Addgene) using the Gateway LR Clonase Enzyme Mix. Lentiviral particles were produced by co-transfecting HEK-293T cells with pMD2G, pMDLg/pRRE and pRSV-Rev plasmids, and EPHB6-encoding lentiviral construct. MDA-Luc cells were transduced with EPHB6-encoding lentiviral particles or mock-transduced with empty vector particles in the presence of 10 µg/ml polybrene (Sigma), and selected with 400 µg/ml hygromycin (Life Technologies). Cells were lysed with the lysis buffer (Promega) and the protein concentration was determined by the Bicinchoninic acid method. Protein concentration was adjusted to 1 µg/µl with the lysis buffer and 10 μl aliquots were mixed with 50 µl of the luciferase assay reagent (Promega). The luminescence signal was quantitated with a Luminometer (Glomax 20/20, Promega, Madison, WI, USA)

### Stable cell lines

Generation of stable MDA-B6, MDA-B6-M and MDA-pc3 cell lines was described previously [15]. Stable EPHB6 knock-downs were generated using lentiviral particles, encoding EPHB6-targeting shRNA-1 (shB6–1; Santa Cruz, sc-39957-V), according to the manufacturer’s instructions. Cells were transduced in the presence of 10 µg/ml polybrene and selected with puromycin (10 µg/ml; Sigma) for 5 days.

To transduce cells with other shRNA constructs (Santa Cruz: shOCT4-1, sc36123-SH; shErk2-1, sc35335-SH; Sigma: shEPHB6-2 (shB6-2), TRCN0000010677; shOCT4-2 TRCN0000004882; shErk2-2, TRCN0000010040) and previously described GFP or RFP constructs [64], procedures were performed as described for the Luciferase assay.

### Confocal microscopy

For colony visualisation, cells forming colonies on glass coverslips were fixed in 4% formaldehyde, incubated for 1 h in blocking/permeabilization solution (1% BSA, 5% horse serum, 0.1% saponin in PBS) and stained with rhodamine phalloidin. Images were captured with an Olympus FV1000 microscope and Olympus Fluoview software. Channels were split and merged, and image intensity was adjusted where required using ImageJ software. For cell–cell contact analysis, cells were blocked for 1 h in 1% BSA with 5% horse serum in PBS and permeabilized with 0.1% saponin. Cells were incubated with mouse anti-EPHB6 (Santa Cruz) or a matching non-specific IgG for 72 h, rinsed three times with PBS and incubated with anti-mouse Alexa Fluor 594 (Life Technologies, # 8890) in 0.1% BSA in PBS for 1 h. Cells were rinsed and stained with anti-ZO-1 Alexa Fluor 488 or a matching non-specific IgG Alexa Fluor 488 (Thermo Fisher, #339188, #53-4714-80). ProLong Gold antifade with DAPI (Life Technologies) was used as a mounting medium in all confocal microscopy experiments. Cells were visualised with a Carl Zeiss LSM 700 confocal microscope. Z-stack frames were acquired using ZEN 2012 software.

### Dataset analysis and code availability

Two microarray gene expression datasets were downloaded: The Cancer Genome Atlas (TCGA; http://tcga-data.nci.nih.gov) and ArrayExpress (http://www.ebi.ac.uk/arrayexpress/) accession E-GEOD-22220 (Buffa Dataset). All available gene expression data were downloaded. Each dataset was analysed separately to avoid problems that could be caused by combining different microarray technologies, measurement types and normalisation techniques. The downloaded TCGA expression data were originally obtained from Agilent G4502A microarrays and measured as a ratio of the amount of expression in the sample to the amount of expression in Stratagene Universal Human Reference RNA. The downloaded EBI expression data were originally obtained from Illumina HumanRef-8 v1 Expression BeadChip microarrays and expressed as log base 2 intensities. To identify a population of TNBC samples, all breast cancer cases in the TCGA and EBI datasets were grouped into three populations. The first population (“ER positive”) included samples with high levels of ER and PR expression. The second population (“HER2 positive”) included cases that expressed high levels of HER2. The third population (“triple-negative”) represented samples with reduced levels of ER, PR and HER2. These populations were visualised in three dimensions (ER, PR and HER2 expression levels) with the R statistical language and environment (http://www.r-project.org), and parameters gradually adjusted, until separation between the populations could no longer be improved. The relevance of these groupings was confirmed by *k*-means clustering of the datasets using Euclidean distance and *k* = 3, and comparison of cluster validity indices for the resultant clusters versus our populations. The *k*-means clustering was performed using built-in functions in R, and the Dunn and Davies–Bouldin indices were calculated using the clv R library (http://cran.r-project.org/package=clv). Both indices indicated that our groupings were superior to the clusters from *k*-means for both datasets. This allowed us to avoid any loss of relevant data that could potentially be associated with the introduction of arbitrarily selected cutoff values for defining the triple-negative population. EPHB6 expression was assessed in normal breast tissue (where available), in all breast cancer samples combined, in the triple-negative population or in all breast cancer cases excluding the triple-negative population, as indicated in figure legends.

This approach was verified by an additional independent analysis, where TNBC population was defined as the group of samples with the lowest 10% of ER, PR and HER2 expression. Analysis of these populations confirmed all original conclusions. Statistical significance of the differences in EPHB6 expression was determined by Wilcoxon rank-sum tests, since there was no guarantee that the expression levels were normally distributed. Scatter plots were generated and regression analysis was performed to examine correlations between expression of EPHB6 and of other molecules of interest. Spearman correlation and *P*-values were calculated using standard functions in the R statistical language and environment (http://www.r-project.org). Boxplots and scatterplots were generated with base R graphics functions.

### Survival analysis

Kaplan–Meier plot was produced for the recurrence-free survival using the online tool KM-plotter (http://kmplot.com/analysis/) and the 2014 version of the database. The lower tertile was used as a cutoff. All other parameters were left as their default options.

### Statistical analysis

For statistical analyses of experimental data, two-tailed Student’s *t*-test or Mann–Whitney *U*-test were used, depending on the comparison of variances. The data met test assumptions and all tests were applied appropriately for each dataset. Sample sizes in all experiments, including animal studies, were selected empirically, with initial estimates based on our experience in similar models. Experiments, including animal studies, were done in a non-blinded fashion. The data are presented as mean ± SD. Statistical significance was defined as *P* < 0.05.

### Additional information

Western blot data were processed using Odyssey, Carestream, Adobe Illustrator and PowerPoint software. Cropping and adjustment of brightness and contrast in Western blot images was done in PowerPoint.

## Electronic supplementary material

Supplemental Material
