# Supplementary material for: EPHB6 augments both development and drug sensitivity of triple-negative breast cancer tumours
Source: Oncogene. 2018 Apr 27;37(30):4073–93. doi: 10.1038/s41388-018-0228-x (PMC6062499; doi:10.1038/s41388-018-0228-x)
Supplement: Supplementary file 1 — Supplemental Material [file 41388_2018_228_MOESM1_ESM.pdf]

## Supplementary Information

# EPHB6 augments both development and drug sensitivity of triple-negative breast cancer tumours

Behzad M. Toosi, Amr El Zawily, Luke Truitt, Matthew Shannon, Odette Allonby, Mohan Babu, John DeCoteau, Darrell Mousseau, Mohsin Ali, Tanya Freywald, Amanda Gall, Frederick S. Vizeacoumar, Morgan W. Kirzinger, C. Ronald Geyer, Deborah H. Anderson, TaeHyung Kim, Alana L. Welm, Peter Siegel, Franco J. Vizeacoumar, Anthony Kusalik, and Andrew Freywald

## Supplementary Figure Legends

**Figure S1.** EPHB6 affects the motility of TNBC cells and their propagation in tumourspheres**.** (**A**) Expression of the EPHB6 mRNA was analysed in 14 TNBC cell lines and 22 breast cancer cell lines of ER+ and HER2+ origins using data from Cancer Cell Line Encyclopedia (CCLE). Statistical significance was computed using the Mann-Whitney *U* test. n.s.- non-significant. (**B**) MDA-pc3, MDA-B6 and MDA-B6-M cells were seeded into 6-well plates ($5 \times 10^5$ cells per well) in triplicates and allowed to form confluent monolayers. A scratch was made in each monolayer with a 100 μl pipette tip. Wells were rinsed with serum-free medium to remove floating cells or cell debris. The same areas of each scratch (4 per scratch) were imaged at the time of scratching (0h) and 12, 24, and 36 h later with an EVOS FL Cell Imaging System microscope at 100 x magnification. The width of scratch in each image was measured using the Adobe Illustrator software. The graph represents a percentage of scratch open at each time point relative to the 0h point. Solid lines in the graph represent mean values. Scale bar, 400 µM. *, $P < 0.05$ for differences between MDA-pc3 and MDA-B6, and between MDA-pc3 and MDA-B6-M; Student's *t*-test. (**C**) Levels of EPHB6 mRNA expression in TNBC cell lines, BT20 and HCC70. RNA from BT20 and HCC70 cells was prepared with a QIAGEN RNeasy Mini Kit (cat # 74104), according to the manufacturer's instructions and EPHB6 mRNA levels were analysed by qPCR. In each replicate, 1 μg of RNA was used in the High Capacity cDNA Reverse Transcription Reaction (Thermo Fisher Scientific, cat # 4368814) by following kit instructions. Taqman Gene Expression Master Mix (Thermo Fisher Scientific, cat # 4369016) and Taqman probes (Thermo Fisher Scientific EPHB6 assay, ID Hs01071144_m1, cat # 4331182) were used to setup qPCR analyses. The graph represents three experiments, each performed in technical triplicates. EPHB6 mRNA expression is

presented in arbitrary units (AU) relative to matching GAPDH controls. (**D**) Expression of the indicated EMT/MET markers was examined in EPHB6-expressing and EPHB6-deficient TNBC cells by Western blotting. (**E**) The indicated cells were propagated in tumourspheres and analysed as in Fig. 5c. Scale bar, 1000 µM. *, $P < 0.05$; Student's *t*-test. (**F**) Representative images of tumourspheres shown at a higher magnification, scale bar, 400 µM. (**G**) EPHB6 expression was analysed in the indicated cell lines by Western blotting. (**H**) EPHB6 expression and silencing are preserved in xenograft models. Xenograft tumours of the indicated origins were allowed to develop in NOD SCID mice for up to 40 days. Mice were terminated, tumours were extracted and EPHB6 expression in tumours was analysed by Western blotting.

Data are shown as means ± SD. All experiments were performed three times. For optimal presentation, individual scratch and tumoursphere images are shown at different brightness and contrast settings.

**Figure S2.** (**A**) EPHB6 augments tumour growth. BT20-NS and BT20-shB6-1 cells were injected into the mammary fat pad region of 4-6 weeks old NOD SCID mice (n = 4; $1.5 \times 10^6$ cells per mouse). Tumour growth was monitored as in Fig. 6a**.** Mice were euthanized on day 31 after injection, tumours were excised, photographed and weighed.

Data are shown as means ± SD. Animals were randomly assigned to all experimental groups. The data represent one of two independent experiments. *, $P < 0.05$; Student's *t*-test.

**Figure S3.** ERK activity is essential for EPHB6 action in TNBC cells. (**A**) ERK phosphorylation was monitored by Western blotting in the indicated cells. (**B**) BT-20 cells were treated for 2 h at 37 ºC with MEK inhibitor, PD0325901 (100 nM), or a matching volume of DMSO and phosphorylation status of ERK kinases was assessed by Western blotting. (**C**)

BT-20 cells were cultured for 72 hours with PD0325901 (100 nM) or a matching volume of DMSO, and OCT4 expression was assessed by Western blotting. (**D**) BT-20 cells were propagated for 7 days in tumourspheres in the presence of PD0325901 (100 nM) or a matching volume of DMSO and cell proliferation was examined as in Fig. 5c. (**E**) MDA-B6-M cells were transduced with additional ERK2-targeting shRNA, shErk2-2, or with non-silencing shRNA as a control (MDA-B6-M-NS). ERK2 expression was assessed by Western blotting. The effect of ERK2 silencing on cell proliferation in tumourspheres was analysed as in Fig. 5c. (**F**) Oct4 expression was analysed in the indicated cells by Western blotting. (**G**) BT-20 cells were transduced with ERK2-targeting shRNAs, shErk2-1 or shErk2-2, individually, or with non-silencing shRNA as a control (BT20-NS). ERK2 silencing was assessed by Western blotting. Quantifications were done as in Fig. 2d. Proliferation of BT20-shErk2-1, BT20-shErk2-2 and BT20-NS cells in tumourspheres was analysed as in Fig. 5c.
Data are shown as means ± SD. Experiments were performed at least three times. *, $P < 0.05$; Mann-Whitney U-test. Scale bars, 1000 µM. For optimal presentation, individual tumoursphere images are shown at different brightness and contrast settings.

**Figure S4.** EPHB6 controls responses in a TNBC patient-derived xenograft. (**A-D**) Cells isolated from a TNBC patient-derived xenograft, HCI-010, were transduced with additional EPHB6-targeting shRNA,  shB6-2, or with non-silencing shRNA as a control (BT20-NS) and analysed by Western blotting for EPHB6 (A), vimentin (B) and OCT4 (C) expression; proliferation of these cells in tumourspheres was analysed as in Fig. 5c (D). (**E**) HCI-010-NS and HCI-010-shB6-2 cells were analyzed by flow cytometry to determine the proportion of aldehyde dehydrogenase 1 (ALDH1)-positive cells. The data represent one of two independent experiments. (**F**) ERK phosphorylation was monitored by Western blotting in the indicated cells. (**G**) HCI-010 cells were transduced with additional ERK2-targeting shRNA,

shErk2-2 and ERK2 silencing was confirmed by Western blotting. Proliferation of the indicated cells in tumourspheres was analysed as in Fig. 5c. (**H**) EPHB6 silencing is maintained in PDX tumours. PDX tumours of the indicated origins were allowed to develop in NOD SCID mice for up to 50 days. Mice were terminated, tumours were extracted and EPHB6 expression was analysed by Western blotting.

Data are shown as means ± SD. Experiments were performed at least three times, until otherwise indicated. *, $P < 0.05$; Student's *t*-test. Scale bars, 1000 µM. For optimal presentation, individual tumoursphere images are shown at different brightness and contrast settings.

**Figure S5.** EPHB6 accelerates growth of TNBC tumours and suppresses their drug resistance. (**A-C**) HCI-010-NS and HCI-010-shB6-2 cells were injected into the mammary fat pad region of 4-6 weeks old NOD-SCID mice (1 x $10^6$ cells per mouse). Mice with tumours were treated with doxorubicin or saline (n = 5 per condition) as in Fig. 8a. Growth rates of saline-treated HCI-010-NS and HCI-010-shB6-2 tumours were compared (A). Doxorubicin-induced reductions in growth of HCI-010-NS and HCI-010-shB6-2 tumours are presented as a percentage relative to matching saline-treated controls (B). Upon termination of the experiment at day 46 following initial cell injections, the excised tumours were photographed and weighed (C). The left graph in panel (C) represents tumour weights in doxorubicin-treated mice as a percentage relative to matching saline-treated controls. Average tumour weights for each group of experimental animals are shown in the right graph.

Data are shown as means ± SD. Animals were randomly assigned to all experimental groups. *, $P < 0.05$; Student's *t*-test or Mann-Whitney U-test. n.s., statistically not significant.

**Figure S6.** Effects of DNA-damaging and proteasome-inhibiting drugs on EPHB6-expressing or EPHB6-deficient TNBC cells. (**A**) MDA-pc3 and MDA-B6-M tumoursphere-producing cells, or MDA-pc3 and MDA-B6-M grown in monolayers were incubated for 24 h with increasing concentrations of doxorubicin or matching volumes of PBS, as indicated. Cells were stained with resazurin, and the cytotoxic effect was measured using a SpectraMax M5 microplate reader (Molecular Devices, LLC., Sunnyvale, CA, USA). Data represent the analysis of three independent experiments performed in triplicates and are shown as a percentage of change in doxorubicin-induced cell killing of MDA-B6-M relative to killing efficiency in MDA-pc3 cells. (**B**) MDA-pc3 and MDA-B6-M tumoursphere-producing cells, or MDA-pc3 and MDA-B6-M grown in monolayers were incubated for 24 h with 160 nM docetaxel or matching volume of DMSO and cell survival was assessed by the MTT assay using a SpectraMax 340PC microplate reader (Molecular Devices). Cell killing was analysed as in (A). (**C**) MDA-pc3 and MDA-B6-M tumoursphere cells were treated for 24 h with the indicated concentrations of a proteasomal inhibitor, bortezomib, or matching volumes of DMSO. Cells were stained with resazurin and cell killing was analysed as in (A).

Data are shown as means ± SD. Experiments were performed at least three times.

*, $P < 0.05$; Student's *t*-test or Mann-Whitney U-test. n.s., statistically not significant.

# Figure S1

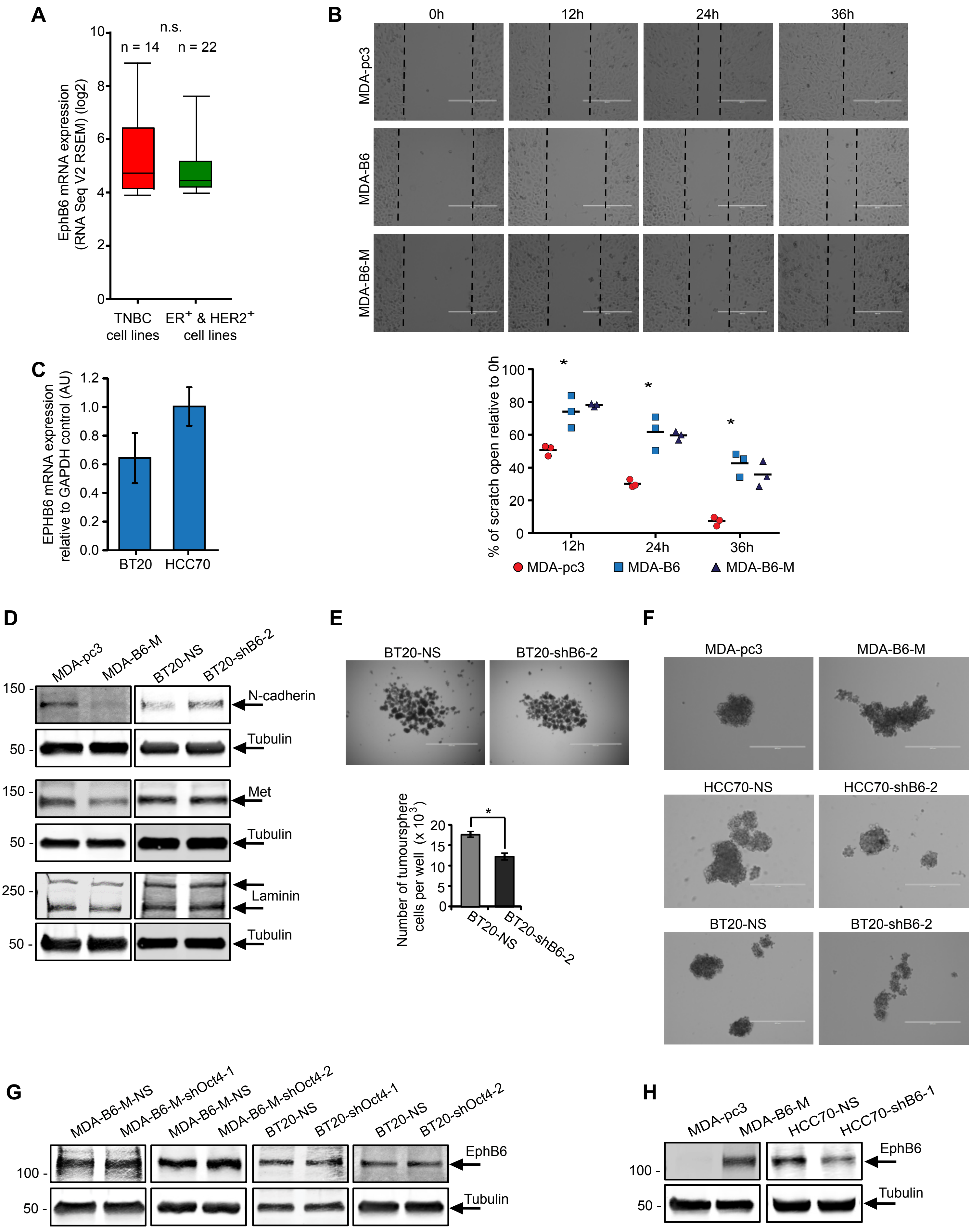
A
n.s.
n = 14
n = 22
EphB6 mRNA expression (RNA Seq V2 RSEM) (log2)
TNBC cell lines
ER+ & HER2+ cell lines
B
0h
12h
24h
36h
MDA-pc3
MDA-B6
MDA-B6-M
% of scratch open relative to 0h
MDA-pc3
MDA-B6
MDA-B6-M
C
EPHB6 mRNA expression relative to GAPDH control (AU)
BT20
HCC70
D
MDA-pc3
MDA-B6-M
BT20-NS
BT20-shB6-2
N-cadherin
Tubulin
Met
Laminin
E
BT20-NS
BT20-shB6-2
Number of tumoursphere cells per well (x 10^3)
F
MDA-pc3
MDA-B6-M
HCC70-NS
HCC70-shB6-2
BT20-NS
BT20-shB6-2
G
MDA-B6-M-NS
MDA-B6-M-shOct4-1
MDA-B6-M-NS
MDA-B6-M-shOct4-2
BT20-NS
BT20-shOct4-1
BT20-NS
BT20-shOct4-2
EphB6
Tubulin
H
MDA-pc3
MDA-B6-M
HCC70-NS
HCC70-shB6-1
EphB6
Tubulin

## Figure S2

**A**

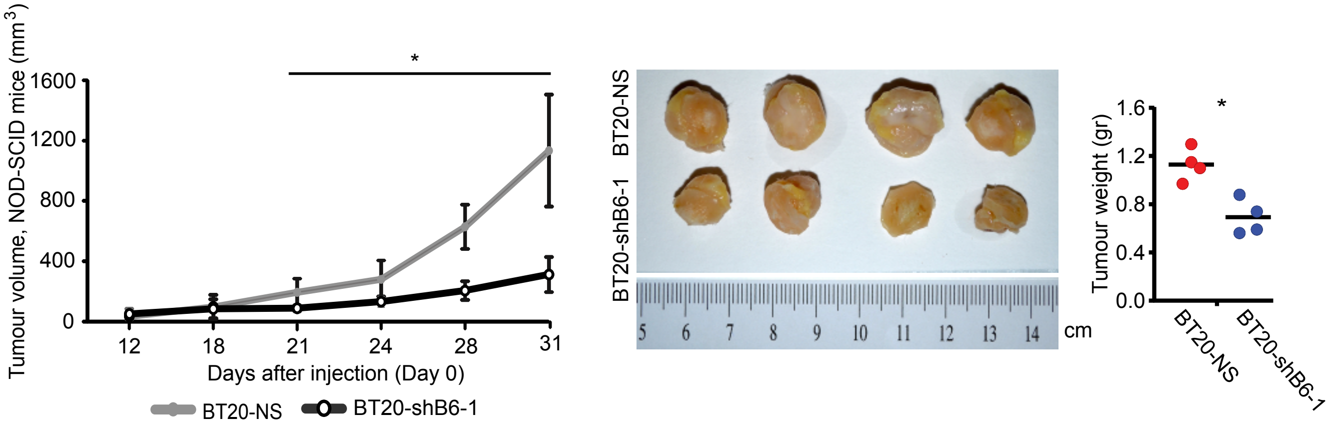
Tumour volume, NOD-SCID mice (mm$^3$)
1600
1200
800
400
0
12
18
21
24
28
31
Days after injection (Day 0)
*
BT20-NS
BT20-shB6-1
BT20-NS
BT20-shB6-1
5 6 7 8 9 10 11 12 13 14 cm
Tumour weight (gr)
1.6
1.2
0.8
0.4
0.0
*
BT20-NS
BT20-shB6-1

**Figure S3**

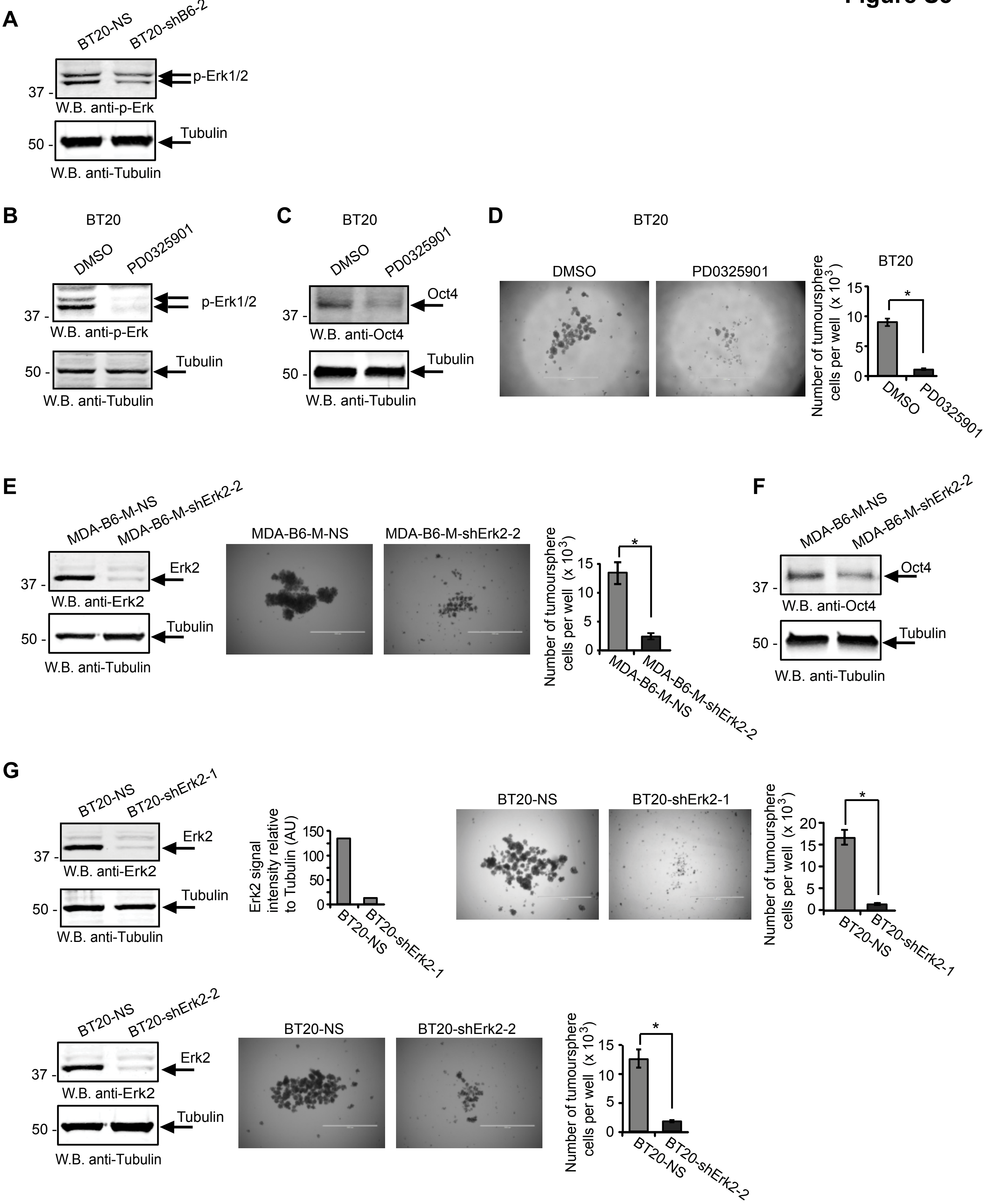

A
BT20-NS
BT20-shB6-2
p-Erk1/2
37 -
W.B. anti-p-Erk
Tubulin
50 -
W.B. anti-Tubulin
B
BT20
DMSO
PD0325901
p-Erk1/2
37 -
W.B. anti-p-Erk
Tubulin
50 -
W.B. anti-Tubulin
C
BT20
DMSO
PD0325901
Oct4
37 -
W.B. anti-Oct4
Tubulin
50 -
W.B. anti-Tubulin
D
BT20
DMSO
PD0325901
BT20
Number of tumoursphere cells per well (x 10^3)
15
10
5
0
*
DMSO
PD0325901
E
MDA-B6-M-NS
MDA-B6-M-shErk2-2
Erk2
37 -
W.B. anti-Erk2
Tubulin
50 -
W.B. anti-Tubulin
MDA-B6-M-NS
MDA-B6-M-shErk2-2
Number of tumoursphere cells per well (x 10^3)
15
10
5
0
*
MDA-B6-M-NS
MDA-B6-M-shErk2-2
F
MDA-B6-M-NS
MDA-B6-M-shErk2-2
Oct4
37 -
W.B. anti-Oct4
Tubulin
50 -
W.B. anti-Tubulin
G
BT20-NS
BT20-shErk2-1
Erk2
37 -
W.B. anti-Erk2
Tubulin
50 -
W.B. anti-Tubulin
Erk2 signal intensity relative to Tubulin (AU)
150
100
50
0
BT20-NS
BT20-shErk2-1
BT20-NS
BT20-shErk2-1
Number of tumoursphere cells per well (x 10^3)
20
15
10
5
0
*
BT20-NS
BT20-shErk2-1
BT20-NS
BT20-shErk2-2
Erk2
37 -
W.B. anti-Erk2
Tubulin
50 -
W.B. anti-Tubulin
BT20-NS
BT20-shErk2-2
Number of tumoursphere cells per well (x 10^3)
15
10
5
0
*
BT20-NS
BT20-shErk2-2

# Figure S4

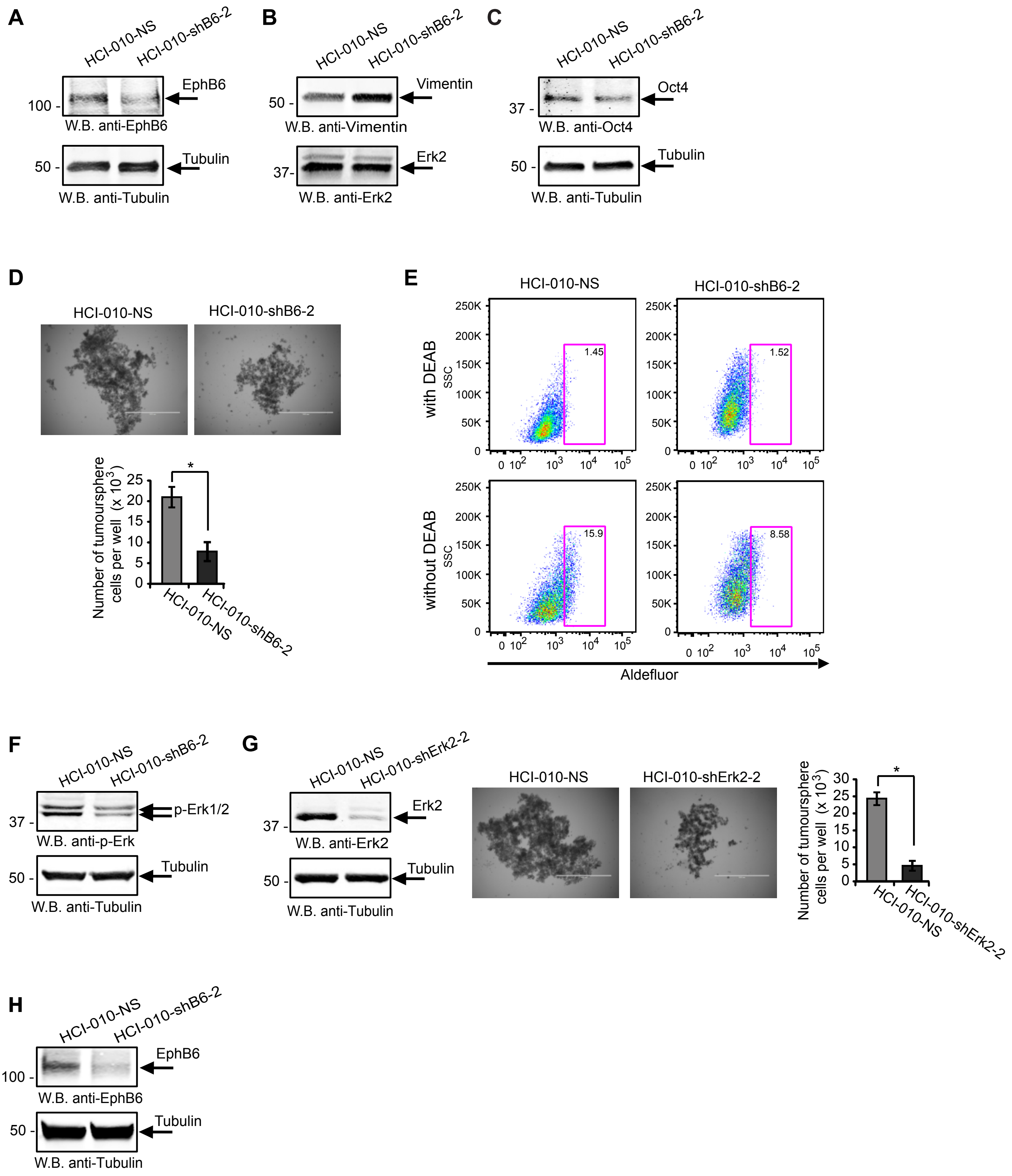
A
HCI-010-NS
HCI-010-shB6-2
EphB6
100 -
W.B. anti-EphB6
Tubulin
50 -
W.B. anti-Tubulin
B
Vimentin
50 -
W.B. anti-Vimentin
Erk2
37-
W.B. anti-Erk2
C
Oct4
37 -
W.B. anti-Oct4
Tubulin
50 -
W.B. anti-Tubulin
D
HCI-010-NS
HCI-010-shB6-2
Number of tumoursphere cells per well (x 10^3)
*
E
HCI-010-NS
HCI-010-shB6-2
with DEAB
without DEAB
SSC
1.45
1.52
15.9
8.58
Aldefluor
F
p-Erk1/2
37 -
W.B. anti-p-Erk
Tubulin
50 -
W.B. anti-Tubulin
G
HCI-010-NS
HCI-010-shErk2-2
Erk2
37 -
W.B. anti-Erk2
Tubulin
50 -
W.B. anti-Tubulin
Number of tumoursphere cells per well (x 10^3)
*
H
HCI-010-NS
HCI-010-shB6-2
EphB6
100 -
W.B. anti-EphB6
Tubulin
50 -
W.B. anti-Tubulin

**Figure S5**

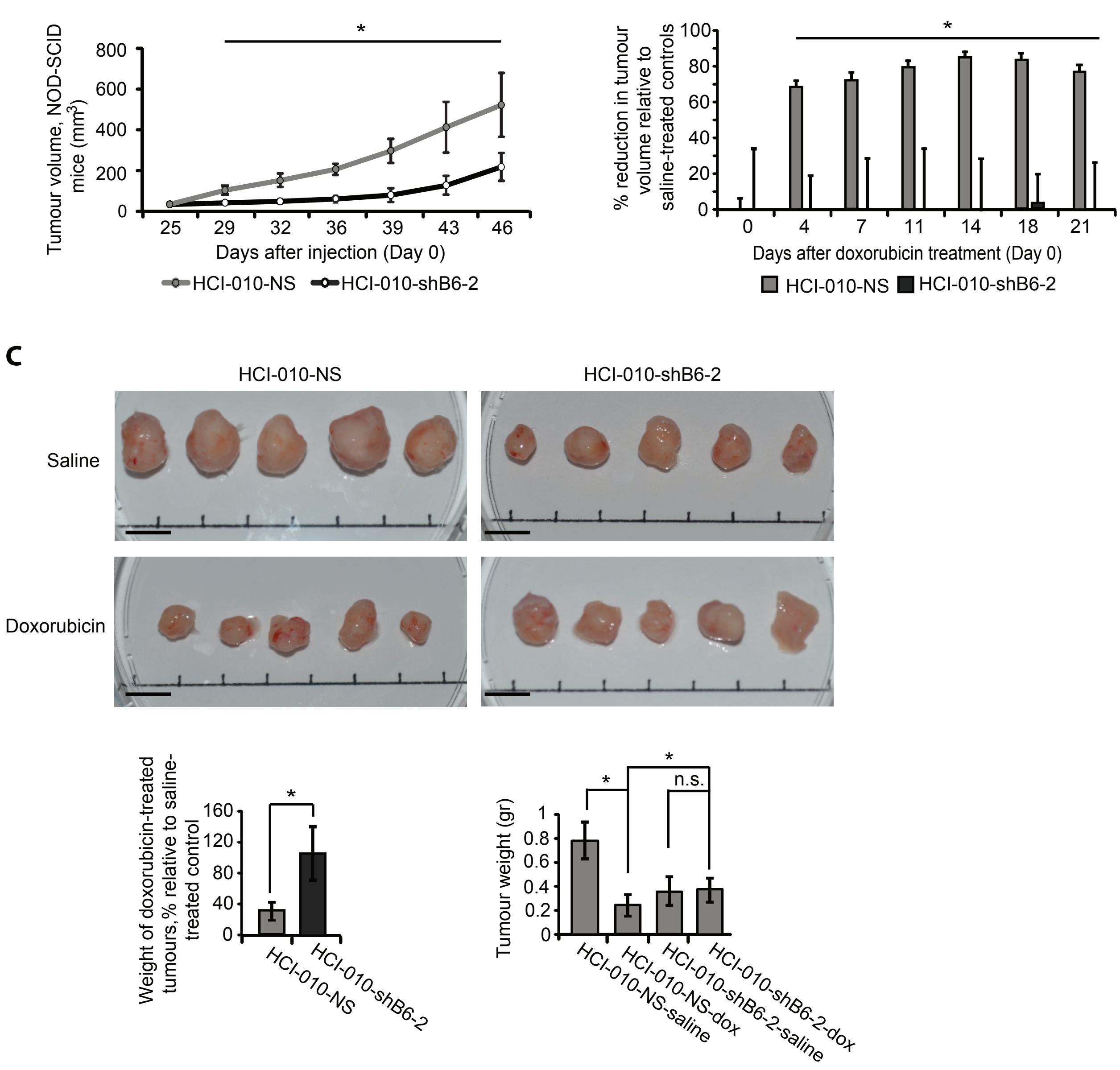
A
Tumour volume, NOD-SCID mice (mm3)
800
600
400
200
0
25
29
32
36
39
43
46
Days after injection (Day 0)
*
HCI-010-NS
HCI-010-shB6-2
B
% reduction in tumour volume relative to saline-treated controls
100
80
60
40
20
0
0
4
7
11
14
18
21
Days after doxorubicin treatment (Day 0)
*
HCI-010-NS
HCI-010-shB6-2
C
HCI-010-NS
HCI-010-shB6-2
Saline
Doxorubicin
Weight of doxorubicin-treated tumours,% relative to saline-treated control
160
120
80
40
0
*
HCI-010-NS
HCI-010-shB6-2
Tumour weight (gr)
1
0.8
0.6
0.4
0.2
0
*
*
n.s.
HCI-010-NS-saline
HCI-010-NS-dox
HCI-010-shB6-2-saline
HCI-010-shB6-2-dox

# Figure S6

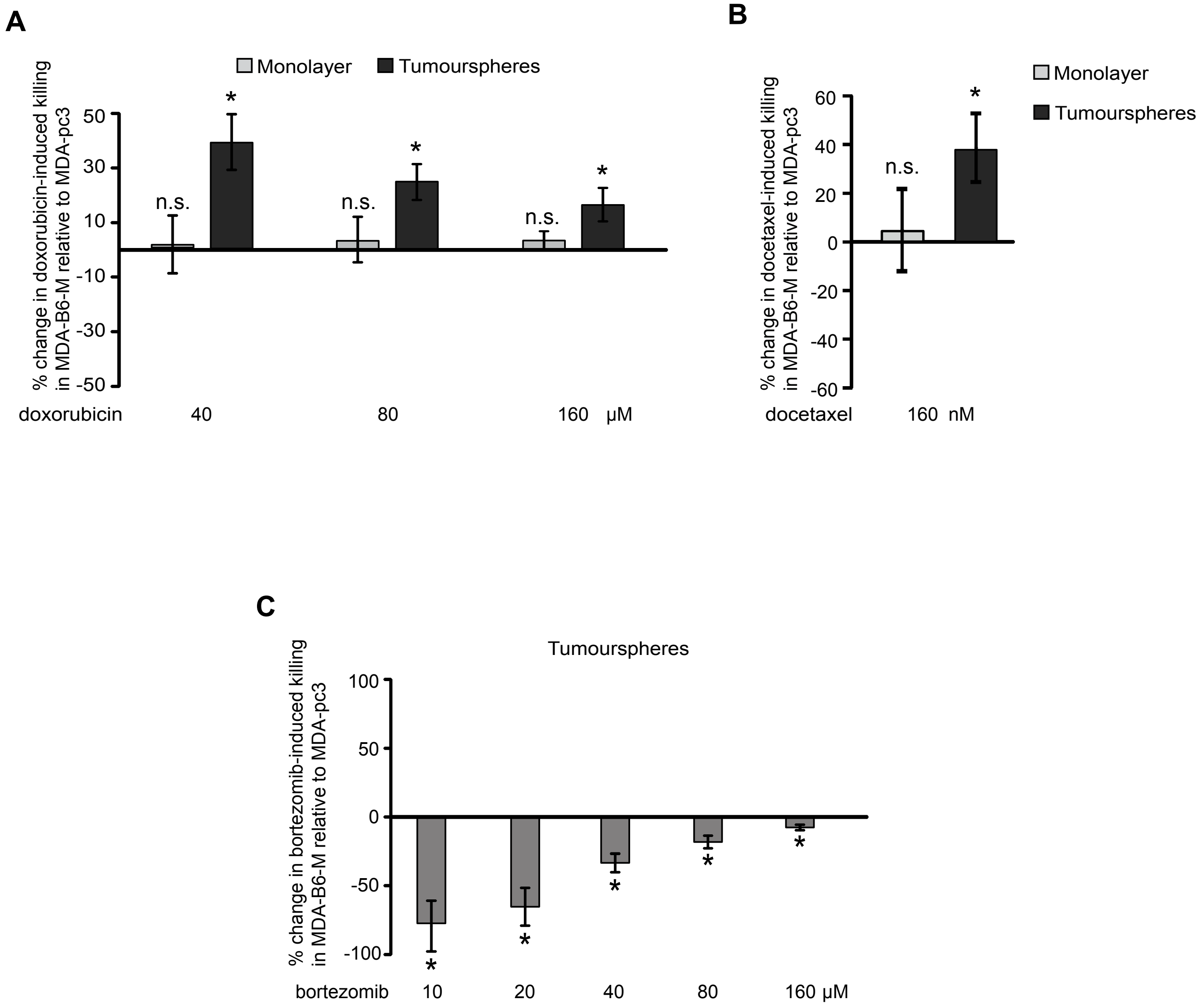
A
Monolayer
Tumourspheres
% change in doxorubicin-induced killing in MDA-B6-M relative to MDA-pc3
50
30
10
-10
-30
-50
n.s.
*
doxorubicin
40
80
160 µM
B
Monolayer
Tumourspheres
% change in docetaxel-induced killing in MDA-B6-M relative to MDA-pc3
60
40
20
0
-20
-40
-60
docetaxel
160 nM
C
Tumourspheres
% change in bortezomib-induced killing in MDA-B6-M relative to MDA-pc3
100
50
0
-50
-100
bortezomib
10
20
40
80
160 µM
